# Proteome Analyses of Soil Bacteria Grown in the Presence of Potato Suberin, a Recalcitrant Biopolymer

**DOI:** 10.1264/jsme2.ME15195

**Published:** 2016-10-28

**Authors:** Amadou Sidibé, Anne-Marie Simao-Beaunoir, Sylvain Lerat, Lauriane Giroux, Vicky Toussaint, Carole Beaulieu

**Affiliations:** 1 Centre SÈVE, Département de Biologie, Université de SherbrookeSherbrooke (QC), J1K 2R1Canada; 2 Horticulture Research and Development Centre, Agriculture and Agri-Food CanadaSaint-Jean-sur-Richelieu (QC), J3B 3E6Canada

**Keywords:** bacterial succession, β-oxidation, lipase, lipid metabolism, metaproteomics

## Abstract

Suberin is a complex lipidic plant polymer found in various tissues including the potato periderm. The biological degradation of suberin is attributed to fungi. Soil samples from a potato field were used to inoculate a culture medium containing suberin as the carbon source, and a metaproteomic approach was used to identify bacteria that developed in the presence of suberin over a 60-d incubation period. The normalized spectral counts of predicted extracellular proteins produced by the soil bacterial community markedly decreased from day 5 to day 20 and then slowly increased, revealing a succession of bacteria. The population of fast-growing pseudomonads declined and was replaced by species with the ability to develop in the presence of suberin. The recalcitrance of suberin was demonstrated by the emergence of auxotrophic bacteria such as *Oscillatoria* on the last days of the assay. Nevertheless, two putative lipases from *Rhodanobacter thiooxydans* (I4WGM2) and *Myxococcus xanthus* (Q1CWS1) were detected in the culture supernatants, suggesting that at least some bacterial species degrade suberin. When grown in suberin-containing medium, *R. thiooxydans* strain LCS2 and *M. xanthus* strain DK 1622 both produced three lipases, including I4WGM2 and Q1CWS1. These strains also produced other proteins linked to lipid metabolism, including fatty acid and lipid transporters and β-oxidation enzymes, suggesting that they participate in the degradation of suberin. However, only the *R. thiooxydans* strain appeared to retrieve sufficient carbon and energy from this recalcitrant polymer in order to maintain its population over an extended period of time.

Suberin is a cell wall-associated biopolymer found in various tissues including tuber periderms. It acts as a lipophilic barrier to protect plant tissues against dehydration, wounding, and pathogen attacks (5). The extensive deposition of suberin in plant cell walls leads to cell death. Cork is a multilayered dead tissue that mainly consists of suberin (46). Suberin is not only produced during plant development, its biosynthesis is also induced by biotic stress and wounding (42).

Suberin is a complex polymer comprising aromatic and aliphatic domains, which are covalently linked by glycerol ester bonds (5). The aromatic domain mainly consists of polyhydroxycinnamates (4, 7) and the aliphatic domain is a fatty acid polyester that shares structural similarities with cutin (5). The aliphatic moiety of suberin is composed of fatty acids, fatty alcohols, and ω-hydroxyfatty acids with chains of up to 30 carbon atoms (14). In the last few decades, research has provided insights into the chemical structure of suberin and its biosynthetic pathways; however, limited information is available on its biodegradation.

Suberin is recalcitrant to microbial degradation (27), a property that explains why cork is the preferred material for wine bottle stoppers. Its slow degradation also means that suberin biomarkers are good tracers for investigating soil organic matter dynamics (16). The turnover of the suberin lipidic fraction was previously estimated to correspond to a soil residence time of more than 30 years (10). The decomposition of suberin is attributed to fungal communities. Some fungi, including plant pathogens such as *Rosellinia desmazieresii* (38), *Rigidoporus lignosus*, and *Phellinus noxius* (36), have the ability to penetrate suberized periderms. Furthermore, esterases exhibiting activity against suberin have been purified from cultures of various fungal genera including *Aspergillus* (13), *Fusarium* (11), and *Coprinopsis* (27). Several suberin-degrading esterases have been identified as cutinases and are active on cutin and suberin (21). A whole genome transcriptome analysis of *Aspergillus nidulans* revealed the main pathways involved in the degradation of suberin: ester hydrolysis, ω-hydroxyfatty acid oxidation, and peroxisomal β-oxidation (33).

The involvement of bacteria in the decomposition of suberin is not well documented. Nevertheless, bacteria exhibiting cutinase activity have been isolated, including members of the genera *Bacillus* (2), *Pseudomonas* (48), and *Thermomonospora* (12). Bacterial cutinases may also be active on suberin because cutinase production is induced by suberin in *Thermomonospora* (12). Evidence has also accumulated to indicate that *Streptomyces scabiei*, the causal agent of potato common scab, degrades potato suberin. The *S. scabiei* genome encodes potential cutinase-encoding genes and one of these, *sub1*, has been shown to be specifically induced in the presence of suberin (25).

In the present study, the soil bacteria of a potato field were grown in medium containing suberin as the carbon source. A proteomic analysis of the secretome was performed in order to identify enzymes and bacterial taxons involved in the degradation of suberin. Since this initial proteomic analysis allowed for the identification of putative lipases from *Rhodanobacter thiooxydans* and *Myxococcus xanthus*, a strain from both species was grown in the presence of suberin and their secretomes were characterized.

## Materials and Methods

### Culture conditions

SM, medium containing suberin as the sole source of carbon, was composed of potato suberin (0.1%) and mineral solution containing (NH_4_)_2_SO_4_ (0.5 g L^−1^), K_2_HPO_4_ (0.5 g L^−1^), MgSO_4_-7H_2_O (0.2 g L^−1^), and FeSO_4_-7H_2_O (0.01 g L^−1^). Suberin in this medium was extracted from potato peels (24).

SM supplemented with nystatin (50 mg L^−1^) and cycloheximide (50 mg L^−1^) was used to grow the soil bacterial community from a potato field in Pont-Rouge (Quebec, Canada). Soil samples (2 g) were used to inoculate 100 mL of SM with antifungal compounds, which was then incubated under shaking (120 rpm) at 30°C for 60 d. After 5-, 10-, 20-, and 30-d incubations, the culture was centrifuged at 3,450×*g* for 20 min and 67 mL of the supernatant was sampled and kept for the proteomic analysis. The pellet was then resuspended in the remaining supernatant and 67 mL of fresh mineral solution was added to the culture. The incubation was then resumed under the same conditions. Supernatants were also sampled after a 60-d incubation.

Bacterial inoculums of *R. thiooxydans* strain LCS2 and *M. xanthus* strain DK 1622 were prepared as follows. Strains LCS2 and DK 1622 were grown with shaking (120 rpm) at 30°C for 5 d in R2A medium (28) or 3 d in CTT (17) broth, respectively. The culture was then centrifuged at 3,450×*g* at 25°C for 10 min and washed twice with sterile distilled water. A sample of this suspension (2 mL) was used to inoculate 100 mL of SM. Bacteria were grown with shaking (120 rpm) at 30°C. After 5-, 10-, and 20-d incubations, a fraction of the culture supernatant was replaced with fresh mineral solution as described above. The 5- and 30-d-old supernatants from the *R. thiooxydans* culture were kept for further proteomic analyses. Regarding *M. xanthus*, sampling was processed on 5- and 25-d-old supernatants, and carried out in two replicates.

Growth curves for *R. thiooxydans* LCS2 and *M. xanthus* DK 1622 were established by inoculating SM to reach 2–3 10^4^ CFU mL^−1^ of bacterial cells in 5 mL and 25 mL, respectively. Cultures were incubated without agitation at 28°C (*R. thiooxydans*) or 30°C (*M. xanthus*) for 30 d. Cultures were sampled periodically and serial dilutions of the samples were spread on R2A-agar (28) or CTT agar (17). Inoculated Petri dishes were placed at 28°C or 30°C for 5 to 7 d before CFU counts. The experiment was performed in four replicates. Control samples were also monitored in SM deprived of suberin.

### Proteomic analysis

EDTA (0.3 mM, final concentration) was added to supernatant samples in order to prevent protein degradation. Supernatant proteins were concentrated 35 times using Centricon (Amicon Ultra-15 Centrifugal Filters 3K). Proteins were subjected to sodium dodecyl sulfate-polyacrylamide gel electrophoresis (10% [w/v] SDS-PAGE) as described by Komeil *et al.* (26). In-gel protein digestion and mass spectrometry were conducted at the Proteomics Platform of the Eastern Quebec Genomics Center (Quebec, Canada) using a quadrupole time-of-flight mass spectrometer (Qq-TOF) (AB Sciex) coupled to HPLC as previously described (26).

### Secretome analysis

MS/MS spectra were analyzed for peptide identification using Mascot (Matrix Science, London, UK; version 2.5.1) to search the URB_3_Bacteria, TAX_*Rhodanobacter*, and TAX_*Myxococcus* databases using previously described search criteria (26). Peptides were grouped into proteins using the Scaffold software program (version Scaffold 4.0.5, Proteome Software, Portland, OR, USA) and protein identification was only considered valid if a 99% ID probability was reached and if at least two unique peptides, in which the cut-offs for peptide thresholds were 95%, were associated with the protein.

The extracellular localization of the protein was obtained using the SignalP (41), Phobius (22), SecretomeP (3), TatP (3), and Tatfind (44) programs. Protein function was predicted using the NCBI, UniProt, KEGG, and COG databases. Since the number of spectra detected for a protein depends on its molecular weight (MW), the number of spectra (SpC) for a protein was normalized by dividing this number by the MW of the corresponding protein to give the normalized spectral count (NSpC).

### Protein diversity

Simpson’s diversity index (1) was used to measure protein function diversity (*Df*) and taxonomic diversity (*Dt*), as follows:

$$Df\;\text{or }Dt=1-\sum_{i=1}^{s}pi^{2}$$

where *pi* is the proportion between NSpC associated with a functional group and total NSpC and the proportion between NSpC associated with a bacterial genus and NSpC for the community for *Df* and *Dt*, respectively.

## Results

### Secretome of a soil bacterial community grown on suberin as the carbon source

**Fig. 1:**
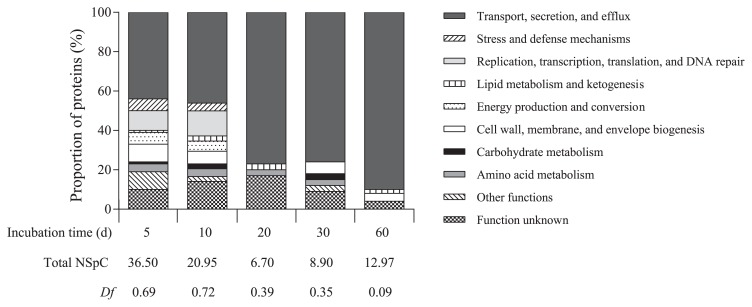
Functional diversity index and distribution into functional groups of predicted extracellular proteins produced by a soil bacterial community grown in minimal medium supplemented with suberin.

A proteomic study was performed in order to investigate the behavior of soil bacteria exposed to potato suberin. Culture medium containing suberin as the carbon source was inoculated with soil and incubated for a 60-d period. Extracellular proteins from culture media were periodically sampled and analyzed. Only a fraction of the culture supernatant was sampled in order to prevent the breakup of potential enzymatic cascades and this fraction was replaced by fresh mineral solution, thereby providing a fresh supply of microelements. Only proteins with a predicted extracellular localization (42% of all proteins) were analyzed further because predicted intracellular proteins may originate from lysed cells that did not multiply on suberin. A total of 244 different extracellular proteins were identified over time (Table S1) and classified into ten functional groups. Fig. 1 shows the distribution of proteins into the functional groups and shows protein diversity and abundance over time.

The number of predicted extracellular proteins markedly decreased from day 5 to day 20, with the NSpC dropping from 36.5 to 6.7. NSpC then slowly increased to reach 13 after a 60-d incubation (Fig. 1). Proteins involved in the functional group “Transport, secretion, and efflux” were the most abundant and their concentrations increased in the culture from day 10 to day 60. At the end of the experimental period, this protein category comprised more than 90% of the supernatant proteins. Accordingly, *Df* continually decreased from 0.69 on day 5 to 0.09 on day 60 (Fig. 1). There were few proteins involved in “Lipid metabolism and ketogenesis” (NSpC=0.00 to 0.34) at any of the sampling times. In this protein category, two putative lipases were identified: I4WGM2 (NSpC=0.11) and Q1CWS1 (NSpC=0.02), which are associated with *R. thiooxydans* and *M. xanthus*, respectively (Table S1).

Most of the proteins identified were associated with a sole bacterial genus (Table S1). When it was possible to assign more than one bacterial genus to a protein, the protein was arbitrarily assigned to the genus associated with the highest NSpC at the relevant sampling date. The secretome analysis revealed a low *Dt* after 5 d of growth (*Dt*=0.18). This diversity index *Dt* increased to 0.69 on day 10 and stabilized at approximatively 0.80 thereafter (0.83, 0.79, and 0.77 on day 20, 30, and 60, respectively). The low *Dt* observed on day 5 may be explained by approximately 90% of the secreted proteins being assigned to *Pseudomonas* species (Table S1). Only two genera produced detectable amounts of extracellular proteins at any of the sampling times examined: *Methylotenera* and *Pseudomonas* (Table 1). However, NSpC assigned to *Pseudomonas* markedly decreased over time, from 32.9 on day 5 to 0.1 on day 60 (Table 1). In contrast, NSpC continuously increased from day 5 to the end of the culture period for three genera: *Bradyrhizobium*, *Variovorax*, and *Ralstonia*. At the end of the culture period, proteins secreted by *Burkholderia* and *Ralstonia* predominated (NSpC of 4.3 and 4.2, respectively). Proteins produced by nine bacterial genera (*Afipia*, *Bacteroides*, *Flavobacterium*, *Hyphomicrobium*, *Niastella*, *Oscillatoria*, *Salinibacter*, *Sphingomonas*, and *Stenotrophomonas*) and by one unclassified bacterium were only detected on day 60 (Table 1).

### Secretomes of *R. thiooxydans* and *M. xanthus* grown on suberin as the sole carbon source

**Fig. 2:**
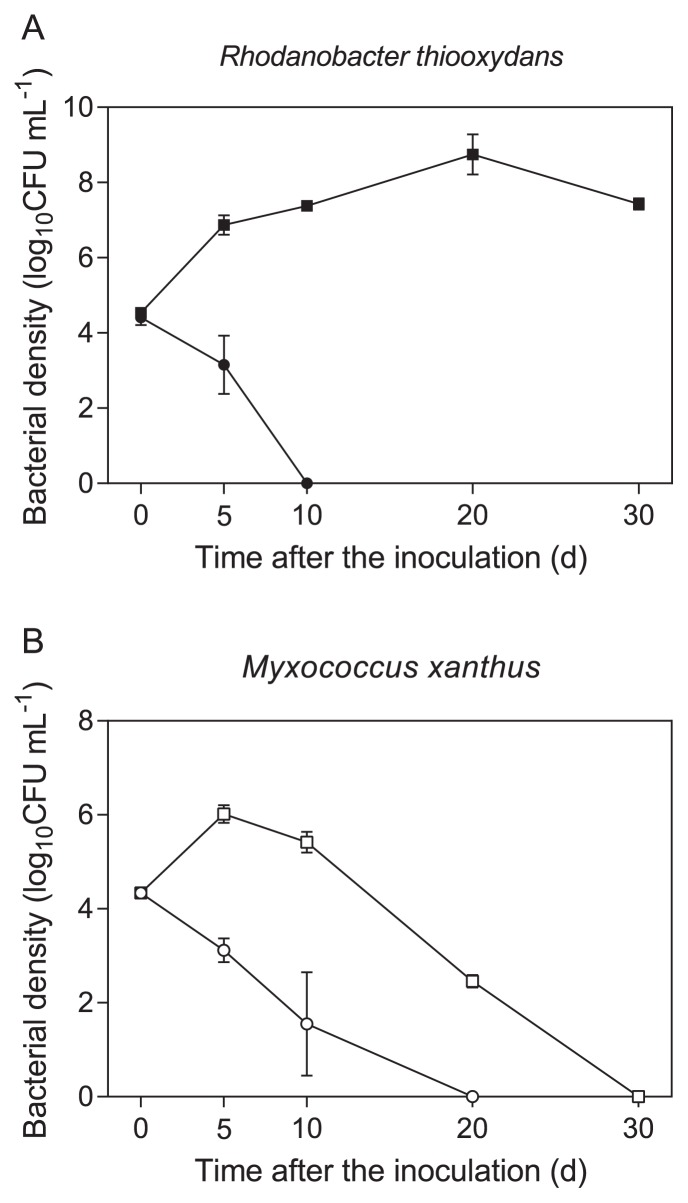
Growth curve of *Rhodanobacter thiooxydans* LCS2 (A) and *Myxococcus xanthus* DK 1622 (B) in minimal medium supplemented with suberin as the sole source of carbon (squares) or not supplemented (circles).

The chemical structure of suberin suggests that esterases such as lipases are involved in its hydrolysis. Since lipases from *R. thiooxydans* and *M. xanthus* were detected in the secretome of the bacterial community, we hypothesized that these bacteria are potential suberin degraders, and, thus, a strain of both species was grown in suberin-containing medium. Fig. 2 shows the growth curves of both strains in suberin-containing medium. The growth of *R. thiooxydans* increased from 0 to 20 d and stabilized at 7.4 log_10_CFU mL^−1^ after a 30-d incubation. In the absence of suberin, growth decreased from 4.5 (day 0) to 3.3 log_10_CFU mL^−1^ on day 5. No viable bacteria were detected on day 10 or thereafter (Fig. 2A). The growth of *M. xanthus* was observed in suberin-containing medium during the first 5 d of growth (from 4.3 on day 0 to 6.0 log_10_CFU mL^−1^ on day 5); however, after 10 d of growth, the bacterial population constantly decreased. In the absence of suberin, *M. xanthus* did not grow and no viable bacteria were detected from day 10 (Fig. 2B).

**Fig. 3:**
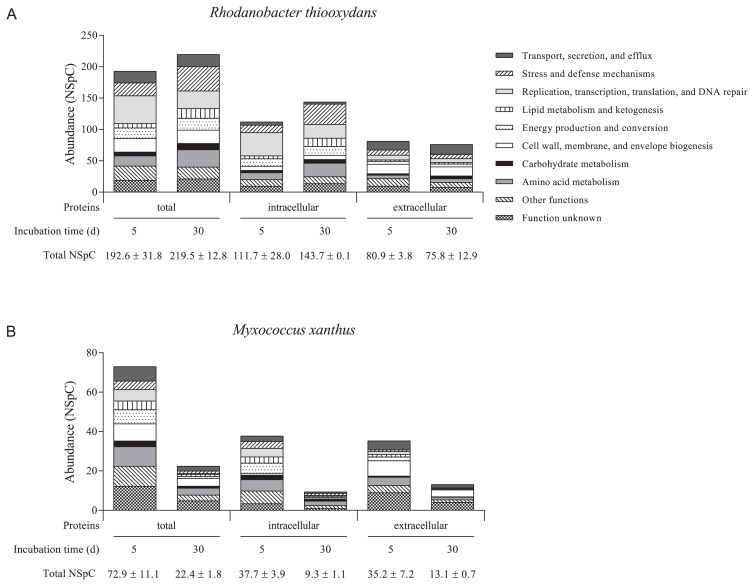
Functional diversity index and distribution into functional groups of proteins produced by *Rhodanobacter thiooxydans* LCS2 (A) and *Myxococcus xanthus* DK 1622 (B) grown in minimal medium supplemented with suberin.

*R. thiooxydans* and *M. xanthus* protein profiles were both examined at two sampling times. A total of 839 proteins for *R. thiooxydans* (Table S2) and 644 proteins for *M. xanthus* (Table S3) were classified into ten functional groups (Fig. 3), and 48% and 27% of these proteins, respectively, were present at both sampling times. In *R. thiooxydans*, the predicted extracellular proteins represented approximately 42% of the identified proteins on day 5 and day 30. The proportion of predicted extracellular proteins was higher in the *M. xanthus* proteome, representing 47% and 60% of all proteins on day 5 and day 25, respectively. Protein abundance (NSpC) was 192.6 and 219.5 on day 5 and day 30, respectively, for *R. thiooxydans* (Fig. 3A). Fewer proteins were detected in the *M. xanthus* supernatant. NSpC decreased from day 5 to day 25 (72.9 and 22.8 on day 5 and 25, respectively; Fig. 3B). Protein distribution within the functional groups showed different profiles depending on the sampling time in the *R. thiooxydans* and *M. xanthus* proteomes (Fig. 3). On day 30, the predominant functional group of *R. thiooxydans* was “Stress and defense mechanisms” (NSpC=38.8), whereas proteins linked to “Replication, transcription, translation, and DNA repair” predominated on day 5 (NSpC=44.4; Fig. 3A). Proteins of unknown function represented an important part of the *M. xanthus* proteome at both sampling times (13% and 9% of total NSpC on day 5 and day 25, respectively), and the proportion of proteins within the “Carbohydrate metabolism” and the “Stress and defense mechanisms” groups decreased by half between day 5 and day 25 (Fig. 3B).

Fifty and 37 of the identified proteins belonged to the “Lipid metabolism and ketogenesis” functional group in *R. thiooxydans* and *M. xanthus*, respectively (Table 2 and 3), including the predicted extracellular lipases, I4WUC2, I4WSC3, and I4WGM2 of *R. thiooxydans* and Q1CWS1 and Q1D5W1 of *M. xanthus*. The lipases I4WGM2 and Q1D5W1 were also detected in the secretome of soil bacteria (Table S1). *R. thiooxydans* proteins in this category were more abundant on day 30 than on day 5, representing 7.0% and 3.6% of all NSpC, respectively. The proportion of predicted intracellular proteins involved in “Lipid metabolism and ketogenesis” increased from 4.2% to 9.0% between day 5 and day 30, whereas the concentration of predicted extracellular proteins in this category remained stable at both sampling times (Fig. 3A). The proportion of proteins linked to “Lipid metabolism and ketogenesis” was more stable in *M. xanthus*, representing 3.1% and 3.9% of the predicted extracellular proteins and 8.3% and 6.2% of the intracellular proteins on day 5 and day 25, respectively (Fig. 3B). Proteins from other functional groups were also potentially linked to lipid metabolism. For example, the proteins I4WDW1, I4WU70, I4WHD4, Q1D009, Q1D3Z8, Q1D5Z6, and Q1DDZ2 were predicted to play a role in lipid binding or transport (Table S2, S3 and 4). Table 4 proposes putative suberin degradation and utilization pathways in *R. thiooxydans* and *M. xanthus*.

## Discussion

While fungi (19, 33) appear to play a role in the degradation of suberin, the involvement of soil bacteria has been poorly documented. In the present study, bacteria from a potato field were exposed to suberin for a 60-d period. The marked decrease observed in the amount of proteins produced in the first 20 days of the culture indicates that suberin is an efficient carbon and energy source for most soil bacteria. *Pseudomonas* has been reported as one of the ten most important genera found in the bulk soil of potato fields (19). Although proteins from the genus *Pseudomonas* were predominant on the first days of the culture, this may reflect the rapid growth of *Pseudomonas* on carbon sources present in soil (29). Proteins from other genera that were abundant in the same bulk soils, such as *Rhodanobacter*, *Sphingomonas*, and *Mucilaginibacter* (19), were not detected in the culture supernatant before at least 30 days of growth. Although the concentration of proteins associated with *Pseudomonas* markedly decreased over time, we cannot rule out the possibility that members of this genus utilize suberin components such as glycerol and lipids. Pseudomonads secreted J0YBC2, a Yce1-like protein (a protein with lipid-binding properties [45]); E2XW23, a long-chain fatty acid transporter; and C3JZ29, a glycerophosphoryl diester phosphodiesterase, enzymes involved in glycerol and lipid catabolism (26). Nevertheless, the constant decrease in *Pseudomonas* protein concentrations suggests that the energy obtained from the suberin substrate did not allow the *Pseudomonas* population to successfully compete with other microorganisms.

While the amount of extracellular proteins markedly decreased in the first 20 days of growth, the secreted proteins originated from more diversified bacterial populations. The increase observed in extracellular protein concentrations from day 20 to day 60 may have resulted from the proliferation of bacterial species more adapted to survive and grow in the presence of a recalcitrant compound such as suberin. These species comprise bacteria that do not depend on suberin as an energy or carbon source (autotrophs), oligotrophs that are capable of growth on very low concentrations of nutrients, and suberin-utilizing bacteria. The emergence of autotrophic bacteria such as *Oscillatoria* and the persistence of an insoluble suberin substrate in culture medium after a 60-d incubation reflect the recalcitrant nature of suberin. A recent study examining the bacterial succession of microorganisms in the rhizosphere of plants grown in sand demonstrated that bacterial diversity declined, with autotrophs becoming dominant due to the scarcity of carbon sources (49).

*Burkholderia* was the genus showing the highest protein concentration at the end of the incubation period. However, this population may be composed of genotypically different subpopulations (50) because the concentration of proteins associated with the genus initially decreased and then gradually increased. Proteins for which concentrations progressively increased over time originated from three bacterial genera: *Bradyrhizobium*, *Ralstonia*, and *Variovorax*. However, the proliferation of these genera was not unequivocally attributed to their ability to use suberin as a carbon source because the proteins identified in this study did not encode enzymes involved in plant cell wall degradation. The retention of *Bradyrhizobium* and *Ralstonia* in the prolonged culture may be due to their oligotrophic nature. McAlister *et al.* (34) isolated *Bradyrhizobium* and *Ralstonia* oligotrophic strains from ultrapure water and showed that some of them were capable of cryptic growth. Recent studies demonstrated that the plant pathogen *Ralstonia solanacearum* degraded hydroxycinnamic acid (31), the main constituent of the suberin aromatic fraction (4).

When *S. scabiei* was grown in the presence of suberin, the vast majority of its extracellular proteins were identified as glycosyl hydrolases (26). Only one glycosyl hydrolase has been found in the secretome of the bacterial community, a xylanase from *S. scabiei*, confirming that residual polysaccharides embedded in suberin are not readily accessible for microbial degradation (26, 39). In this study, at the end of the culture period, *Df* was low and most proteins identified belonged to the functional group “Transport, secretion, and efflux”. Other metaproteomic studies on bacterial populations living on plant surfaces have also mainly identified proteins linked to nutrient transport (23).

The two predicted lipases detected in the community belonged to the genera *Myxococcus* and *Rhodanobacter*, respectively. While *Myxococcus* is known to colonize various plants (15) and produce cell surface enzymes exhibiting esterase activity (43), *Rhodanobacter* has been identified as one of the predominant genera colonizing potato tubers (19). Members of this genus have been detected in soils in which potatoes, but not barley have been cultivated (20).

*R. thiooxydans* and *M. xanthus* were both grown in a pure culture on potato suberin. In contrast to *Rhodanobacter*, which expanded or maintained its population during the entire incubation period, *Myxococcus* ceased to grow after 10 d. However, only a small fraction of the secretome appeared to be dedicated to lipid metabolism in both bacteria. A similar situation was observed when *S. scabiei* was grown on suberin (26) or when *Bacillus* was grown on cutin (2), a polymer bearing structural similarities to suberin. The analysis of *Myxococcus* and *Rhodanobacter* proteomes revealed that both bacteria may possess not only the tools to depolymerize the aliphatic fraction of suberin, but also the ability to bind suberin and assimilate fatty acids (Table 4). The abilities of *R. thiooxydans* and *M. xanthus* to grow on fatty acids as carbon source have been previously reported (6, 28). The detection in this study of a fructose-1,6-bisphosphatase (I4WC19) and phosphoenolpyruvate carboxykinase (Q1DCV2), which are dedicated to gluconeogenesis, is an indication that fatty acids contribute to the growth of *R. thiooxydans* and *M. xanthus* in suberin-containing medium.

I4WDW1 and Q1D009, abundant proteins found in the secretomes of *R. thiooxydans* and *M. xanthus*, respectively, are putative lipid-binding proteins that may facilitate the accessibility of the bacterial enzymatic battery to suberin. The lipases of *Rhodanobacter* and of *Myxococcus* (Table 4) may release fatty acids from suberin by hydrolyzing the ester bonds linking the components of suberin. Q1D4F3 and Q1CWS1 are putative patatin-like phospholipases that have been shown to exhibit activity on monoacylglycerol (35), a depolymerization product of the potato periderm (30). Furthermore, *M. xanthus* produced a feruloyl esterase (Q1D458), and these enzymes have been proposed to cleave the aliphatic fraction of suberin from the aromatic moiety (26). The degradation products released by esterases and lipases, which may then be assimilated as proteins involved in lipid or long-chain fatty acid transport (Table 4), were detected in both bacterial species. In *Escherichia coli*, outer membrane-bound long-chain fatty acid transporters act as channels, allowing fatty acid assimilation (8). These long-chain fatty acid transporters function in conjunction with inner membrane-associated fatty acyl CoA synthetases that activate these compounds to CoA thioesters, thereby rendering this entry process unidirectional (8); I4WFP4 and I4WB55 or Q1D9B8, Q1D855, and Q1CYM5 may play a similar role in *R. thiooxydans* and *M. xanthus*, respectively. Since enzymes predicted to achieve the four steps of β-oxidation (37) were detected (Table 4), long-chain fatty acids may actually serve as carbon sources for *Rhodanobacter* and *M. xanthus*. While *Rhodanobacter* β-oxidation proteins were more abundant after a prolonged incubation (total NSpC of 1.39 and 4.18 on day 5 and day 30, respectively), the amount of these enzymes decreased in *Myxococcus* (total NSpC of 1.19 and 0.33 on day 5 and day 25, respectively). After 10 d of growth in the presence of suberin as the carbon source, the *Myxococcus* population constantly declined, which may indicate that the amount of fatty acids released from suberin did not provide sufficient energy for bacterial proliferation. The presence of several carboxylases (Q1CYB4, Q1DDA0, Q1D0B9, Q1DOT9, Q1D555, Q1DDA2, and Q1D8V2) in the *Myxococcus* proteome also supports this hypothesis. Carboxylation represents a cellular biosynthetic mechanism to replenish tricarboxylic acid cycle intermediates used in biosynthetic pathways (9).

In contrast, the presence of an acetoacetate decarboxylase (I4WM96) that catalyzes the production of acetone in the *R. thiooxydans* proteome suggests that acetate yielded by β-oxidation needs to be converted into acetone in order to prevent acid accumulation and toxicity (40). In an excess of carbon, several bacteria have been shown to exhibit the ability to convert products of the β-oxidation process into storage material such as polyhydroxyalkanoates (PHA) (32). Since enzymes providing precursors for PHA biosynthesis (I4WKH2, I4WKH3, and I4WB09), enzymes directly involved in PHA biosynthesis (I4WUD4, I4VZY3, I4WKE0, and I4WKE1) (32) as well as phasin (I4WMY1), a surface protein associated with PHA granules (47), have been detected in *R. thiooxydans* suberin medium, PHA biosynthesis by *R. thiooxydans* is likely to occur.

The production of several proteins involved in lipid metabolism strongly suggests that *R. thiooxydans* and *M. xanthus* have the capacity to obtain carbon from the aliphatic fraction of potato suberin. However, *M. xanthus* did not appear to retrieve sufficient carbon from this substrate to ensure its proper and prolonged growth on suberin; however, we cannot exclude the possibility that long-chain fatty acids are toxic to *M. xanthus*. Nevertheless, the production of lipid-binding proteins and cell surface enzymes with esterase activity (43) may confer this predatory soil bacterium with the ability to compete for space and nutrients on the potato periderm surface. The predilection of *Rhodanobacter* for the potato tuber environment (20) has not been explained; however, its capacity to at least partly degrade potato periderm constituents may represent a competitive advantage over numerous bacteria present in the rhizosphere. While this study confirmed that suberin is recalcitrant to degradation by several soil bacterial populations, the involvement in this process of heterotroph soil bacteria that predominate after a prolonged incubation in suberin culture medium warrants further investigation.

## Supplementary Information



## Figures and Tables

**Table 1:** Abundance of predicted extracellular proteins (NSpC) within diverse bacterial genera identified in suberin medium inoculated with potato field soil

Bacterial genus	Incubation time (d)				

	5	10	20	30	60
*Acidovorax*	—a	0.15	—	0.48	0.25
*Acinetobacter*	2.04	2.39	1.83	0.25	—
*Afipia*	—	—	—	—	0.24
*Azospirillum*	—	—	—	0.10	—
*Bacteroides*	—	—	—	—	0.02
*Bordetella*	—	0.19	—	—	—
*Bradyrhizobium*	—	0.05	0.16	0.29	0.44
*Burkholderia*	—	8.71	0.01	0.68	4.27
*Comamonas*	—	—	0.61	0.43	—
*Cupriavidus*	—	0.07	—	0.43	1.14
*Elusimicrobium*	—	—	—	0.03	—
*Flavobacterium*	—	—	—	—	0.02
*Hyphomicrobium*	—	—	—	—	0.06
*Methylobacterium*	—	—	0.65	0.09	0.19
*Methylotenera*	0.35	0.21	0.23	0.19	0.10
*Mucilaginibacter*	—	—	—	0.06	0.10
*Myxococcus*	—	—	0.02	—	—
*Niastella*	—	—	—	—	0.02
*Oscillatoria*	—	—	—	—	0.06
*Patulibacter*	—	1.49	1.08	0.08	—
*Polynucleobacter*	—	0.35	—	—	—
*Pseudomonas*	32.90	7.10	1.43	1.33	0.11
*Ralstonia*	—	0.08	0.16	3.68	4.17
*Rhizobium*	—	—	0.05	0.10	0.10
*Rhodanobacter*	—	—	—	0.03	0.35
*Rhodopseudomonas*	—	—	0.13	—	0.36
*Rickettsia*	—	—	—	0.11	0.33
*Salinibacter*	—	—	—	—	0.02
*Serratia*	1.21	—	—	—	—
*Sphingomonas*	—	—	—	—	0.05
*Stenotrophomonas*	—	—	—	—	0.06
*Streptomyces*	—	—	—	0.08	—
*Variovorax*	—	0.15	0.25	0.44	0.49
Unclassified bacteria	—	—	—	—	0.02

a —: protein not detected or protein identification not considered valid.

**Table 2:** *Rhodanobacter thiooxydans* proteins involved in lipid metabolism and ketogenesis found after a 5- or 30-d incubation

Uniprot accession number	Putative function	Abundance (NSpC)^a^		Predicted cellular localization^b^

		5 d	30 d	
I4VIC2	acyl carrier protein	—	1.44	I
I4VRU7	3-hydroxyacyl-CoA dehydrogenase	—	0.28	I
I4VV45	YdiF, acetate-CoA transferase	—	0.13	I
I4VY74	3-oxoacyl-ACP reductase	—	0.07	I
I4VZY3	acetoacetyl-CoA reductase	—	0.06	E
I4W091	acyl-CoA dehydrogenase domain-containing protein	0.04	—	I
I4WAL3	3-hydroxydecanoyl-[acyl-carrier-protein] dehydratase	—	0.08	E
I4WAL4	β-ketoacyl-[ACP] synthase I	0.12	0.43	E
I4WAZ2	acetoacetyl-CoA thiolase	0.66	0.66	E
I4WB09	3-hydroxybutyryl-CoA dehydratase	0.13	—	I
I4WB55	long-chain fatty acyl CoA ligase	—	0.07	I
I4WBU8	acetyl-coenzyme A synthetase	0.11	0.12	I
I4WBY7	3-hydroxybutyrate dehydrogenase	—	0.09	E
I4WBZ6	acetyl-CoA acetyltransferase	0.13	0.07	E
I4WCN8	GpsA, glycerol-3-phosphate dehydrogenase	0.13	0.07	E
I4WDA0	acyl-CoA dehydrogenase	0.04	0.25	I
I4WE25	fatty acid binding protein	0.15	0.09	I
I4WE72	branched-chain alpha-keto acid dehydrogenase subunit E2	0.38	0.22	I
I4WEJ2	FabZ, 3-hydroxyacyl-[acyl-carrier-protein] dehydratase	—	0.29	I
I4WEK0	acetyl-coenzyme A carboxylase carboxyl transferase	—	0.04	I
I4WFP4	fatty acyl-CoA synthetase	0.16	0.05	E
I4WGM2	lipase	0.14	0.09	E
I4WIC3	acetyl-CoA acetyltransferase	0.23	0.62	I
I4WIC4	3-hydroxyacyl-CoA dehydrogenase	0.20	0.94	I
I4WJC3	polyhydroxyalkanoate depolymerase	0.07	—	I
I4WKE0	poly(R)-hydroxyalkanoic acid synthase, class III, PhaC subunit	0.14	0.05	I
I4WKE1	poly(R)-hydroxyalkanoic acid synthase subunit	0.05	—	E
I4WKH0	3-oxoacyl-[acyl-carrier-protein] synthase	0.12	0.21	I
I4WKH1	acyl carrier protein	0.11	—	I
I4WKH2	3-ketoacyl-[acyl-carrier-protein] reductase	0.20	0.04	E
I4WKH3	malonyl-CoA-acyl carrier protein transacylase	0.14	0.31	E
I4WKH4	3-oxoacyl-[acyl-carrier-protein] synthase	0.16	0.13	I
I4WKP8	acyl-CoA dehydrogenase	—	0.04	I
I4WM78	acyl-CoA dehydrogenase domain-containing protein	—	0.12	I
I4WM94	acyl-CoA dehydrogenase	—	0.06	E
I4WM95	acyl-CoA dehydrogenase	—	0.03	I
I4WM96	acetoacetate decarboxylase	0.20	0.13	E
I4WMY1	phasin	1.86	5.43	I
I4WP46	trans-2-enoyl-CoA reductase	0.11	0.25	I
I4WPL0	enoyl-CoA hydratase	0.19	0.20	I
I4WPU1	acyl-CoA dehydrogenase	—	0.05	E
I4WR42	acyl carrier protein	0.10	0.35	I
I4WR77	enoyl-CoA hydratase	0.08	—	I
I4WSC3	lipase	0.08	0.20	E
I4WSG8	acyl-CoA dehydrogenase	0.37	1.02	I
I4WU86	acyl-CoA thiolesterase	—	0.16	I
I4WUC2	lipase	—	0.07	E
I4WUD4	acetoacetyl-CoA reductase	0.17	—	E
I4WZ09	YdiF, acetate-CoA transferase	0.13	—	I
M4NHA9	acyl-CoA dehydrogenase	—	0.36	I

a Data are the mean of two replicates.

b E: extracellular; I: intracellular.

c —: protein not detected or protein identification not considered valid.

**Table 3:** *Myxococcus xanthus* proteins involved in lipid metabolism and ketogenesis after a 5- or 25-d incubation

Uniprot accession number	Putative function	Abundance (NSpC)^a^		Predicted cellular localization^b^

		5 d	25 d	
Q1CWS1	lipase	0.31	0.21	E
Q1D4F3	patatin-like phospholipase	0.35	0.13	I
Q1CYB4	acetyl co-enzyme A carboxylase carboxyltransferase	0.37	0.16	E
Q1D030	acetyl-coenzyme A synthetase	0.19	0.06	I
Q1D5V0	acetyl-CoA acetyltransferase	0.33	0.10	I
Q1D9B8	long-chain-fatty-acid-CoA ligase	0.20	—	I
Q1D233	3-hydroxyacyl-CoA dehydrogenase	0.17	—	E
Q1D5Y2	carboxyl transferase domain protein	0.19	—	I
Q1D009	YceI-like family protein	0.27	0.14	E
Q1D3D6	acyl-CoA dehydrogenase	0.13	0.12	I
BKT	β-ketothiolase	0.17	—	I
Q1D5U4	acyl-CoA dehydrogenase	0.10	—	I
Q1D984	acetyl-coenzyme A synthetase	0.06	—	I
Q1DDA0	propionyl-CoA carboxylase	0.07	0.02	I
Q1DFT0	3-oxoacyl-[acyl-carrier protein] reductase	0.21	—	I
Q1D0B9	acetyl-CoA carboxylase	0.10	—	I
Q1D340	malonyl CoA-acyl carrier protein transacylase	0.11	—	I
Q1D5U1	3-hydroxyacyl-CoA dehydrogenase	0.18	—	I
Q1D5U2	enoyl CoA dehydratase	0.13	—	I
Q1D4E4	acyl-CoA dehydrogenase	0.07	—	I
Q1D0T9	acetyl CoA carboxylase	0.03	—	I
Q1D555	acetyl-coenzyme A carboxylase carboxyl transferase	0.06	—	I
Q1D234	acetyl-CoA acetyltransferase	0.05	—	I
Q1CZK4	enoyl-[acyl-carrier-protein] reductase	0.07	—	E
Q1DDA2	propionyl-CoA carboxylase	0.04	—	I
Q1D343	3-oxoacyl-[acyl-carrier-protein] synthase	0.07	—	E
Q1D964	acyl-CoA hydrolase	0.08	—	I
Q1CYM5	medium-chain fatty acid-CoA ligase	0.03	—	I
Q1D5Y4	3-hydroxybutyryl-CoA dehydratase	0.05	—	I
Q1D8V2	acetyl-coenzyme A carboxylase carboxyl transferase	0.05	—	I
Q1D5Y1	acyl-CoA dehydrogenase	0.04	—	I
Q1D5W1	patatin-like phospholipase	0.01	—	E
Q1D5V2	3-oxoacid CoA-transferase	0.07	—	I
Q1D855	long-chain-fatty-acid-CoA ligase	0.02	—	I
Q1D566	acyltransferase	0.02	—	I
Q1CZW5	acyl-CoA dehydrogenase	0.02	0.02	I
Q1D003	β-ketothiolase	0.02	0.13	I
A0A0H4WWQ8	acyl-CoA dehydrogenase	0.02	—	I

a Data are the mean of two replicates.

b E: extracellular; I: intracellular.

c —: protein not detected or protein identification not considered valid.

**Table 4:** Putative suberin degradation and utilization pathways in *Rhodanobacter thiooxydans* and *Myxococcus xanthus*

Biological process	Predicted protein function	Uniprot accession number of identified proteins	

		*R. thiooxydans*	*M. xanthus*
**Suberin depolymerization**			
Suberin adhesion	lipid-binding protein	I4WDW1	Q1D009
Depolymerization of the suberin fatty acid polyester structure	lipase	I4WSC3, I4WGM2, I4WUC2^a^	Q1CWS1, Q1D4F3, Q1D5W1^b^
	feruloyl esterase		Q1D548
**Fatty acid utilization**			
Lipid cell entry	lipid transport	I4WU70	Q1D3Z8^b^, Q1D5Z6^b^, Q1DDZ2^b^
	long-chain fatty acid transport	I4WHD4	Q1CWS0
Fatty acid catabolism			
CoA activation	fatty acyl CoA synthetase	I4WFP4	
	long-chain fatty acyl CoA ligase	I4WB55^a^	Q1D9B8^b^, Q1D855^b^
	medium-chain fatty acyl CoA ligase		Q1CYM5^b^
β-oxidation	acyl-CoA dehydrogenase	I4WP46, I4WSG8, I4WDA0, I4WKP8^a^, I4WM94^a^, I4WM95^a^, I4WPU1^a^, I4WM78^a^, M4NHA9^a^, I4W091^b^	Q1D3D6, Q1D4E4^b^, Q1D5Y1^b^, Q1CZW5, A0A0H4WWQ8
	enoyl-CoA hydratase	I4WR77^b^, I4WPL0	Q1D5U2^b^
	3-hydroxyacyl-CoA dehydrogenase	I4WIC4, I4VRU7^a^	Q1D5U1^b^, Q1D233^b^
	acetyl-CoA acetyltransferase	I4WBZ6, I4WIC3	Q1D5VO, Q1D234^b^, BKT^b^, Q1D003^a^
Regulation of the fatty acid utilization process	fatty acid-binding protein	I4WE25	
	acyl CoA hydrolase		Q1D964^b^

a Proteins detected only on day 30 (*R. thiooxydans*) or day 25 (*M. xanthus*).

b Proteins detected only on day 5.
