# Supplementary material for: Proteome Analyses of Soil Bacteria Grown in the Presence of Potato Suberin, a Recalcitrant Biopolymer
Source: Microbes Environ. 2016 Oct 28;31(4):418–26. doi: 10.1264/jsme2.ME15195 (PMC5158114; doi:10.1264/jsme2.ME15195)
Supplement: Supplementary file 1 [file 31_418_s1.pdf]

Table S1 Proteins with predicted extracellular localization produced by soil bacterial community during growth in minimal medium supplemented with suberin, from 5 to 60 days

UniProt or UniRef accession number	Bacterial species	Putative function	Abundance (NSpC)				
			5 d	10 d	20 d	30 d	60 d
**Amino acid metabolism**							
A3KZ66 [a,b]	*Pseudomonas* spp.	Pvds-regulated endoprotease	-[c]	0.67	0.80	0.73	-
C3K3P0 [b]	*Pseudomonas* spp.	dihydroxy-acid dehydratase	0.03	-	-	-	-
C3K6V7 [b]	*Pseudomonas* spp.	protease	0.15	-	-	-	-
C3K907	*Pseudomonas fluorescens*	protease	0.08	-	-	-	-
E1T7C4	*Burkholderia* sp.	ArgE, acetylornithine deacetylase	-	0.04	-	-	-
F2LE35	*Burkholderia gladioli*	protease	-	0.12	-	-	-
J2YKE9	*Pseudomonas* sp.	dipeptidase	0.06	-	-	-	-
P34816	*Pseudomonas syringae*	tryptophan synthase	0.11	-	-	-	-
**Carbohydrate metabolism**							
A3ML77 [a,b]	*Burkholderia* spp.	enolase	-	0.11	-	-	-
C3K5I7	*Pseudomonas fluorescens*	malate:quinone oxidoreductase	0.03	-	-	-	-
C9ZE95	*Streptomyces scabiei*	xylanase A	-	-	0.08	-	-
UPI000289064F	*Pseudomonas* sp.	malic enzyme	0.29	0.09	-	-	-
**Cell wall, membrane and envelope biogenesis**							
A6BTB9 [a,b]	*Yersinia* spp.	peptidoglycan-associated lipoprotein	0.22	-	-	-	-
A8F2R2 [b]	*Rickettsia* spp.	peptidoglycan-associated lipoprotein	-	-	-	0.11	0.33
A9W4H1 [b]	*Methylobacterium* spp.	BamA	-	-	-	-	0.03
A9Z1A5	*Acinetobacter baumannii*	outer membrane protein A	0.19	-	-	-	-
B1FWC4 [b]	*Burkholderia* spp.	peptidoglycan-associated lipoprotein	-	0.53	-	-	-
B2JGA1 [b]	*Burkholderia* spp.	peptidoglycan-associated lipoprotein	-	0.61	-	-	-
C3JYS2 [b]	*Pseudomonas* spp.	TolB	0.13	0.04	-	-	-
C3K1L1	*Pseudomonas fluorescens*	BamB	0.20	-	-	-	-
C3K324	*Pseudomonas fluorescens*	LptD	0.05	-	-	-	-
C3KBX8 [b]	*Pseudomonas* spp.	OmpA family-lipoprotein	0.33	-	-	-	-
C3KDM6 [b]	*Pseudomonas* spp.	phosphatidylserine decarboxylase	0.06	-	-	-	-
E2XZ64	*Pseudomonas fluorescens*	SurA	0.06	-	-	-	-
G8M7G2	*Burkholderia* sp.	TolB	-	0.16	-	-	-
J0YEJ9 [b]	*Pseudomonas* spp.	peptidoglycan-associated lipoprotein	2.11	1.11	-	-	-
J2M1D5 [b]	*Pseudomonas* spp.	outer membrane protein H1	0.10	-	-	-	-
L7HFX4 [b]	*Pseudomonas* spp.	BamD	0.10	-	-	-	-
O85417	*Pseudomonas caricapapayae*	outer membrane lipoprotein I	-	-	-	0.56	-
UPI00028991C8	*Pseudomonas extremaustralis*	flagellin	0.78	-	-	-	-

**Energy production and conversion**							
B1FT18 [b]	*Burkholderia* spp.	PQQ-dependent dehydrogenase	-	0.12	-	-	-
B1FV46 [b]	*Burkholderia* spp.	malate dehydrogenase	-	0.34	-	-	-
C3K121 [b]	*Pseudomonas* spp.	phosphoenolpyruvate synthase	0.11	-	-	-	-
C3K1E7 [b]	*Pseudomonas* spp.	ATP synthase, gamma chain	0.13	-	-	-	-
C3K6M5 [b]	*Pseudomonas* spp.	succinate dehydrogenase flavoprotein subunit	0.09	-	-	-	-
C3KBL8	*Pseudomonas fluorescens*	aerobic glycerol-3-phosphate dehydrogenase	0.17	-	-	-	-
C3KCP3	*Pseudomonas fluorescens*	acetyltransferase	0.30	0.18	-	-	-
C3KE78	*Pseudomonas fluorescens*	azurin	0.31	-	-	-	-
I2BM47 [b]	*Pseudomonas* spp.	electron transfer flavoprotein, beta subunit	0.46	0.08	-	-	-
J1IL09 [b]	*Pseudomonas* spp.	UDP-glucose 6-dehydrogenase	0.08	-	-	-	-
**Lipid metabolism and ketogenesis**							
A9IIX3 [a,b]	*Bordetella* spp.	acetoacetyl-CoA reductase	-	0.19	-	-	-
C3JZ29 [b]	*Pseudomonas* spp.	glycerophosphoryl diester phosphodiesterase	0.07	-	-	-	-
C3K743 [b]	*Pseudomonas* spp.	3-hydroxydecanoyl-[acyl-carrier-protein] dehydratase	0.16	-	-	-	-
I4WGM2	*Rhodanobacter thiooxydans*	lipase	-	-	-	-	0.11
J0YBC2 [b]	*Pseudomonas* spp.	Yce1-like protein	-	0.14	-	-	-
Q1CWS1	*Myxococcus xanthus*	lipase	-	-	0.02	-	-
**Replication, transcription, translation and DNA repair**							
A2W9A3 [a,b]	*Burkholderia* spp.	elongation factor Ts	-	0.10	-	-	-
A4SUU7 [a,b]	*Polynucleobacter necessarius*	elongation factor Tu	-	0.35	-	-	-
A4VHN7 [a,b]	*Pseudomonas* spp.	50S ribosomal protein L16	0.27	-	-	-	-
A4VHP0 [a,b]	*Pseudomonas* spp.	50S ribosomal protein L14	0.15	-	-	-	-
A5VXR4 [b]	*Pseudomonas* spp.	30S ribosomal protein S5	0.61	0.17	-	-	-
A9ADK4 [a,b]	*Burkholderia* spp.	50S ribosomal protein L24	-	0.27	-	-	-
B2JHS8 [b]	*Burkholderia* spp.	LacI, transcriptional regulator	-	0.17	-	-	-
B2T724 [b]	*Burkholderia* spp.	50S ribosomal protein L17	-	0.13	-	-	-
B2T750 [b]	*Burkholderia* spp.	50S ribosomal protein L4	-	0.17	-	-	-
C1DKN6 [b]	*Pseudomonas* spp.	30S ribosomal protein S11	0.43	-	-	-	-
C3JXX2 [b]	*Pseudomonas* spp.	ATP-dependent RNA helicase	0.06	-	-	-	-
C3K2V7 [b]	*Pseudomonas* spp.	50S ribosomal protein L15	0.33	-	-	-	-
C3K3H0 [b]	*Pseudomonas* spp.	RNA helicase	0.03	-	-	-	-
C3KC10	*Pseudomonas fluorescens*	LemA, virulence regulator	0.22	-	-	-	-
C3KCS0	*Pseudomonas fluorescens*	50S ribosomal protein L25	0.18	-	-	-	-
C3KE70 [b]	*Pseudomonas* spp.	50S ribosomal protein L9	0.27	0.20	-	-	-
C5A9H1	*Burkholderia glumae*	30S ribosomal protein S14	-	0.50	-	-	-

E2XL76 [b]	*Pseudomonas* spp.	GTP-binding protein	0.08	-	-	-	-
E7PK79	*Pseudomonas syringae*	30S ribosomal protein S9	0.27	-	-	-	-
I2BR39	*Pseudomonas fluorescens*	ribosomal protein L25	0.33	-	-	-	-
K8R288	*Burkholderia* sp.	transcription accessory protein	-	0.08	-	-	-
**Stress and defense mechanisms**							
A0K6C2 [a,b]	*Burkholderia* spp.	FtsH, metalloprotease	-	0.04	-	-	-
C3K1W9 [b]	*Pseudomonas* spp.	thioredoxin reductase	0.21	-	-	-	-
C3K275	*Pseudomonas fluorescens*	DnaK	0.20	0.07	-	-	-
C3KB07 [b]	*Pseudomonas* spp.	peroxidase	0.08	-	-	-	-
C3KDG0 [b]	*Pseudomonas* spp.	RecA	0.21	-	-	-	-
E2XRC5	*Pseudomonas fluorescens*	thiol peroxidase	0.22	-	-	-	-
E2XY77	*Pseudomonas fluorescens*	hyperosmotically inducible periplasmic protein	0.71	-	-	-	-
E2XY89 [b]	*Pseudomonas* spp.	FtsH, metalloprotease	0.05	-	-	-	-
I2BQB7 [b]	*Pseudomonas* spp.	cold-shock DEAD box protein A	0.07	-	-	-	-
I4L9S5	*Pseudomonas synxantha*	DnaK	-	0.13	-	-	-
**Transport, secretion and efflux**							
A0K2W2 [a,b]	*Burkholderia* spp.	amino acid/amide ABC transporter	-	0.14	-	-	-
A1TUU8 [a,b]	*Acidovorax* spp.	monosaccharide ABC transporter	-	0.15	-	-	-
A1WC85 [a,b]	*Acidovorax* spp.	porin	-	-	-	0.17	0.25
A2VY88 [a,b]	*Burkholderia* spp.	cation/multidrug efflux pump	-	0.02	-	-	-
A5V8N4 [a]	*Sphingomonas wittichii*	TonB-dependent receptor	-	-	-	-	0.02
A8GAT7 [a]	*Serratia proteamaculans*	metal ABC transporter	0.16	-	-	-	-
A8GB28 [a,b]	*Serratia* spp.	amino acid transporter	0.09	-	-	-	-
A8GCJ4 [a]	*Serratia proteamaculans*	porin	0.34	-	-	-	-
A8GH34 [b]	*Serratia* spp.	cationic amino acid ABC transporter	0.11	-	-	-	-
A9VYY1 [b]	*Methylobacterium* spp.	porin	-	-	-	-	0.05
B0KQ05 [b]	*Pseudomonas* spp.	sulfate ABC transporter	0.24	-	-	-	-
B1FY32 [b]	*Burkholderia* spp.	OmpC family porin	-	0.18	-	-	0.15
B1G0N8	*Burkholderia graminis*	porin	-	-	-	-	0.19
B1GB98	*Burkholderia graminis*	porin	-	-	-	0.20	0.48
B1LV39	*Methylobacterium radiotolerans*	porin	-	-	-	-	0.10
B1ZFK7 [b]	*Methylobacterium* spp.	porin	-	-	0.30	-	-
B2JH03 [b]	*Burkholderia* spp.	monosaccharide-transporting ATPase	-	0.26	-	-	-
B2JK19 [b]	*Burkholderia* spp.	extracellular amino acid-binding receptor	-	1.50	-	-	-
B2JM60	*Burkholderia* spp.	porin	-	-	-	-	0.08
B2T4D9	*Burkholderia phytofirmans*	extracellular amino acid-binding protein	-	0.07	-	-	-

B2T6X5	*Burkholderia phytofirmans*	dipeptide-binding protein	-	0.07	-	-	-
B2U879	*Ralstonia pickettii*	OmpA/MotB domain protein	-	-	-	0.65	0.70
B2UDU1	*Ralstonia pickettii*	porin	-	0.08	0.11	1.08	1.03
B2UFC4 [b]	*Ralstonia* spp.	porin	-	-	-	0.27	0.23
B5WCV4 [b]	*Burkholderia* spp.	amino acid ligand-binding receptor	-	0.10	-	-	-
B5WIR3	*Burkholderia* sp.	cationic amino acid ABC transporter	-	0.29	-	-	-
B7S235	*unclassified Gammaproteobacteria*	TonB-dependent receptor	-	-	-	-	0.02
C3JXZ7 [b]	*Pseudomonas* spp.	histidine-binding periplasmic protein	0.11	-	-	-	-
C3JYK8	*Pseudomonas fluorescens*	RND efflux transporter	-	0.17	-	-	-
C3JYX7 [b]	*Pseudomonas* spp.	branched-chain amino acid ABC transporter	0.07	-	-	-	-
C3JZ77 [b]	*Pseudomonas* spp.	maltose-binding protein	0.07	-	-	-	-
C3JZG0	*Pseudomonas fluorescens*	ferric siderophore receptor	0.03	-	-	-	-
C3K0T0	*Pseudomonas fluorescens*	porin A	3.14	0.14	-	-	-
C3K187 [b]	*Pseudomonas* spp.	imelysin	0.13	-	-	-	-
C3K1B9	*Pseudomonas fluorescens*	metal ABC transporter	0.28	-	-	-	-
C3K1N2 [b]	*Pseudomonas* spp.	SecD	0.04	-	-	-	-
C3K3C7 [b]	*Pseudomonas* spp.	glycine betaine/L-proline ABC transporter	0.12	-	-	-	-
C3K3T8 [b]	*Pseudomonas* spp.	putrescine ABC transporter	0.23	-	-	-	-
C3K3Z3 [b]	*Pseudomonas* spp.	ABC transporter	0.07	-	-	-	-
C3K5B7	*Pseudomonas fluorescens*	porin	0.14	-	-	-	-
C3K5V1	*Pseudomonas fluorescens*	OprE3	0.40	-	-	-	-
C3K717 [b]	*Pseudomonas* spp.	amino acid transporter	0.44	-	-	-	-
C3K7C1 [b]	*Pseudomonas* spp.	SecB	0.22	-	-	-	-
C3K7I2	*Pseudomonas fluorescens*	TonB-dependent receptor	0.25	-	-	-	-
C3K8P0 [b]	*Pseudomonas* spp.	nucleoside-binding protein	0.13	-	-	-	-
C3K8Z6	*Pseudomonas fluorescens*	branched amino acid ABC transporter	0.60	-	-	-	-
C3K914	*Pseudomonas fluorescens*	ABC transporter	0.31	-	-	-	-
C3KB06	*Pseudomonas fluorescens*	iron transporter	0.53	-	-	-	-
C3KBL7 [b]	*Pseudomonas* spp.	glutamate/aspartate ABC transporter	0.27	0.15	-	-	-
C3KBT7 [b]	*Pseudomonas* spp.	sulfate ABC transporter	0.16	-	-	-	-
C3KDU7 [b]	*Pseudomonas* spp.	ABC transporter	0.38	-	-	-	-
C3KEB0	*Pseudomonas fluorescens*	amino acid ABC transporter	1.08	-	-	-	-
C3KEC2	*Pseudomonas fluorescens*	OmpA-like	0.62	-	-	-	-
D0S6Y6	*Acinetobacter calcoaceticus*	DcaP-like	0.53	0.53	0.43	0.17	-
D5H5Q2	*Salinibacter ruber*	TonB-dependent receptor	-	-	-	-	0.02
D8D960	*Comamonas testosteroni*	OmpA/MotB	-	-	0.61	0.43	-
D8G164	*Oscillatoria* sp.	TonB receptor	-	-	-	-	0.06

D8JEE3 [b]	*Acinetobacter* spp.	porin	-	0.11	0.11	-	-
D8JLF9	*Acinetobacter* sp.	porin	0.39	0.43	0.43	-	-
D8NXA2	*Ralstonia solanacearum*	porin	-	-	-	0.72	0.74
E2T0T6	*Ralstonia* sp.	porin	-	-	0.05	0.44	0.51
E2XKC2	*Pseudomonas fluorescens*	type I secretion outer membrane protein	0.07	-	-	-	-
E2XKL7 [b]	*Pseudomonas* spp.	branched-chain amino acid ABC transporter	0.13	-	-	-	-
E2XLH2 [b]	*Pseudomonas* spp.	dipeptide ABC transporterer	0.05	-	-	-	-
E2XMS6 [b]	*Pseudomonas* spp.	OmpH	0.37	-	-	-	-
E2XN23	*Pseudomonas fluorescens*	type I secretion membrane fusion protein	0.12	-	-	-	-
E2XW23	*Pseudomonas fluorescens*	long-chain fatty acid transporter	0.04	-	-	-	-
E2Y0H8	*Pseudomonas fluorescens*	porin	0.29	-	-	-	-
E5C8U6 [b]	*Bacteroides* spp.	TonB-linked outer membrane protein	-	-	-	-	0.02
F0KK22	*Acinetobacter calcoaceticus*	porin D	0.13	0.15	0.06	-	-
F6KQ92	*Pseudomonas costantinii*	OprF	0.35	-	-	-	-
G3A5T6	*Ralstonia syzygii*	OmpA	-	-	-	0.54	0.63
G7Z7T4	*Azospirillum lipoferum*	OmaA	-	-	-	0.10	-
G8M678 [b]	*Burkholderia* spp.	extracellular solute-binding protein	-	0.43	-	-	-
G8M683 [b]	*Burkholderia* spp.	OmpC	-	0.13	-	-	-
G8M6G8	*Burkholderia* sp.	OmpA/MotB family protein	-	0.6	-	-	-
G8M893	*Burkholderia* sp.	porin	-	-	-	0.17	0.56
G8MAY7	*Burkholderia* sp.	porin	-	-	-	0.11	0.36
G8MB24 [b]	*Burkholderia* spp.	branched chain amino acid ABC transporter	-	0.07	-	-	-
G8TGU8	*Niastella koreensis*	TonB-dependent receptor G	-	-	-	-	0.02
H0E3A2 [a,b]	*Patulibacter* spp.	phosphate binding protein	-	1.49	1.08	0.08	-
H1KP99	*Methylobacterium extorquens*	porin	-	-	0.22	-	-
H1S518	*Cupriavidus basilensis*	HlyD	-	0.07	-	-	-
H1SI50	*Cupriavidus basilensis*	porin	-	-	-	-	0.18
H1Y2T7	*Mucilaginibacter paludis*	OmpA/MotB domain protein	-	-	-	0.06	0.10
I0KRP1	*Stenotrophomonas maltophilia*	TonB-dependent receptor	-	-	-	-	0.06
I2BKL0 [b]	*Pseudomonas* spp.	OprD	0.10	-	-	-	-
I2BLJ9	*Pseudomonas fluorescens*	porin	-	0.45	-	-	-
I2BPB1	*Pseudomonas fluorescens*	FpvA	0.09	-	-	-	-
I2BVR7	*Pseudomonas fluorescens*	OprD	0.38	-	-	-	-
I2BWR7	*Pseudomonas fluorescens*	TagQ	0.34	-	-	-	-
I2BWZ9	*Pseudomonas fluorescens*	NLPA family lipoprotein	0.39	-	-	-	-
I2IFR8 [b]	*Burkholderia* spp.	porin	-	-	-	-	0.48
I3AHW1	*Serratia plymuthica*	outer membrane protein A	0.29	-	-	-	-
I4K2Q7 [b]	*Pseudomonas* spp.	OprB	0.20	-	-	-	-

I4KG72 [b]	*Pseudomonas* spp.	amino acid ABC transporter	0.29	-	-	-	-
I4KV15	*Pseudomonas synxantha*	TonB-dependent receptor	0.15	-	-	-	-
I4MZT9	*Pseudomonas* sp.	LivK	0.43	-	-	-	-
I4VVL8 [b]	*Rhodanobacter* spp.	TonB-dependent receptor	-	-	-	0.03	0.06
I4W1F0	*Rhodanobacter spathiphylli*	TonB-dependent receptor	-	-	-	-	0.07
I4W299 [b]	*Rhodanobacter* spp.	PilQ	-	-	-	-	0.12
I5D4U3	*Burkholderia terrae*	OmpA/MotB domain-containing protein	-	0.43	-	-	0.52
J2SD86	*Flavobacterium* sp.	TonB-linked outer membrane protein,	-	-	-	-	0.02
J2T7G6	*Variovorax* sp.	porin	-	-	0.20	0.24	0.24
J2TZX3 [b]	*Pseudomonas* spp.	OprD family porin	-	-	0.06	-	-
J2Z2M0 [b]	*Pseudomonas* spp.	peptidoglycan-associated lipoprotein	0.24	0.19	0.08	-	-
J3CB80	*Variovorax* sp.	porin	-	0.10	0.05	0.20	0.25
J3CWR3	*Bradyrhizobium* sp.	porin	-	0.05	0.16	0.29	0.44
J3DTD1	*Pseudomonas putida*	OprD family porin	-	0.15	0.10	0.04	0.06
J3GVG6	*Pseudomonas* sp.	OprD family porin	-	0.13	0.04	-	-
K0HTW4	*Acidovorax* sp.	OmpA/MotB	-	-	-	0.32	-
K8P9Z0	*Afipia broomeae*	porin	-	-	-	-	0.24
K8R697	*Burkholderia* sp.	porin	-	-	-	-	0.15
K8R8N3	*Burkholderia* sp.	OmpC family porin	-	-	-	0.05	0.32
K8RAU9	*Burkholderia* sp.	OmpC family porin	-	0.13	0.10	0.15	0.53
K8RQJ6	*Burkholderia* sp.	porin	-	-	-	-	0.46
K9AC78	*Acinetobacter baumannii*	Omp38	0.39	0.58	0.45	0.08	-
K9ACC8	*Acinetobacter baumannii*	DcaP-like	0.15	0.38	0.21	-	-
K9CQK5	*Acinetobacter baumannii*	transporter, Ompp1/FadL/TodX family	-	0.09	0.06	-	-
L2EK35 [b]	*Cupriavidus* spp.	porin	-	-	-	0.43	0.96
L7H9X2	*Pseudomonas fluorescens*	branched-chain amino acid ABC transporter	0.35	-	-	-	-
L7HLG1	*Pseudomonas fluorescens*	porin	0.76	-	-	-	-
Q213J7	*Rhodopseudomonas palustris*	Omp2b	-	-	0.13	-	0.36
Q46WM6	*Ralstonia eutropha*	porin	-	-	-	-	0.33
Q4FCM8	*Acinetobacter* sp.	outer membrane protein A	0.16	-	-	-	-
Q5QQ59	*Pseudomonas fluorescens*	OprE3	0.49	-	-	-	-
Q6FE98	*Acinetobacter* sp.	porin	0.10	-	-	-	-
Q985C3	*Rhizobium loti*	porin	-	-	0.05	0.10	0.10
UPI0002885851	*Pseudomonas* sp.	LivK	0.63	-	-	-	-
UPI00028A1D9E	*Sphingomonas* sp.	TonB-dependent receptor	-	-	-	-	0.03
UPI00029AA93B	*Pseudomonas geniculata*	TonB-dependent receptor	-	-	-	-	0.05
**Other functions**							
A0KAZ1 [a,b]	*Burkholderia* spp.	cell division factor	-	0.10	-	-	-
A4VLW4 [a,b]	*Pseudomonas* spp.	adenylosuccinate lyase	0.06	-	-	-	-

A6VCJ6 [a]	*Pseudomonas aeruginosa*	polyribonucleotide nucleotidyltransferase	0.14	-	-	-	-
B2KBE7	*Elusimicrobium minutum*	Smc	-	-	-	0.03	-
C3K1M9 [b]	*Pseudomonas* spp.	inositol monophosphatase	0.13	-	-	-	-
C3K1U2	*Pseudomonas fluorescens*	aminotransferase	0.10	-	-	-	-
C3K5Y0 [b]	*Pseudomonas* spp.	NADP(H)-dependent aldo-keto reductase	0.08	-	-	-	-
C3KD06	*Pseudomonas fluorescens*	amidase	0.04	-	-	-	-
C3KEC6 [b]	*Pseudomonas* spp.	adenylate kinase	0.22	-	-	-	-
E2XPE0 [b]	*Pseudomonas* spp.	peptidyl-prolyl cis-trans isomerase	0.58	0.38	-	-	-
E2XX99	*Pseudomonas fluorescens*	iron-regulated protein	0.21	-	-	-	-
I2BTN8 [b]	*Pseudomonas* spp.	chromosome partitioning protein	0.20	-	-	-	-
I2BZ33 [b]	*Pseudomonas* spp.	YeaG	0.24	-	-	-	-
UPI00028A32F2	*Pseudomonas extremaustralis*	VacJ family lipoprotein	0.31	-	-	-	-
**Function unknown**							
A6UXA9 [a,b]	*Pseudomonas* spp.		0.08	-	-	-	-
A2VTE3 [a,b]	*Burkholderia* spp.		-	0.14	-	-	-
B7L1B2 [b]	*Methylobacterium* spp.		-	-	0.12	0.09	-
C3JYK6	*Pseudomonas fluorescens*		-	0.87	-	-	-
C3K3H1	*Pseudomonas fluorescens*		0.29	-	-	-	-
C3K3V2	*Pseudomonas fluorescens*		0.53	-	-	-	-
C3KBL3 [b]	*Pseudomonas* spp.		0.22	-	-	-	-
C3KED5	*Pseudomonas fluorescens*		0.35	-	-	-	-
C6WZ97	*Methylotenera mobilis*		0.19	0.14	0.17	0.12	0.10
D7DNB6	*Methylotenera* sp.		0.16	0.07	0.07	0.07	-
D8JLH9 [b]	*Acinetobacter* spp.		-	0.13	0.09	-	-
D8JYJ3	*Hyphomicrobium denitrificans*		-	-	-	-	0.06
E2XJW5	*Pseudomonas fluorescens*		0.10	-	-	-	-
E2XLQ8	*Pseudomonas fluorescens*		0.38	0.29	-	-	-
E2XUK8	*Pseudomonas fluorescens*		-	0.47	-	-	-
G4MGY8 [b]	*Burkholderia* spp.		-	0.59	-	-	-
I2BNE9 [b]	*Pseudomonas* spp.		0.11	-	-	-	-
I2BVK9	*Pseudomonas fluorescens*		-	0.60	-	-	-
I2BWV2 [b]	*Pseudomonas* spp.		0.44	0.13	-	-	-
J8V5Q9	*Pseudomonas putida*		-	0.11	0.33	-	-
UPI000288EA66	*Pseudomonas extremaustralis*		0.13	-	-	-	-
UPI000289F004	*Pseudomonas extremaustralis*		0.10	-	-	-	-

[a] This protein could be assigned to more than one bacterial genus. The genus mentioned was arbitrarily chosen as it represented the most abundant genus at the sampling date.
[b] This protein could be assigned to more than one species of the genus mentioned.
[c] Protein not detected or protein identification not considered valid.

Table S2 Proteins produced by *Rhodanobacter thiooxidans* LCS2 during growth in minimal medium supplemented with suberin at day 5 and day 30

Uniprot accession number	Putative function	Abundance (NSpC[a])	
		5 days	30 days
**Amino acid metabolism**			
**I4VHY4**[b]	methylisocitrate lyase	0.16	0.23
**I4VI38**	cysteine synthase	-[c]	1.23
**I4VNT3**	isovaleryl-CoA dehydrogenase	0.07	-
**I4VP75**	glutamine synthetase	-	0.07
I4VUT5	pyrroline-5-carboxylate reductase	-	0.07
**I4VWA7**	Xaa-Pro aminopeptidase	0.04	-
**I4W1V5**	anthranilate phosphoribosyltransferase	-	0.04
**I4W2U0**	asparaginase	-	0.14
I4W3N8	HtpX, protease	-	0.05
**I4W5U6**	N-acetylornithine carbamoyltransferase	0.26	-
**I4W668**	aspartate-semialdehyde dehydrogenase	0.08	-
**I4W6F3**	histidine ammonia-lyase	-	0.04
I4W6N2	peptidyl-prolyl cis-trans isomerase	-	0.09
**I4W9W4**	cysteine synthase	3.41	7.35
I4WAC2	peptidyl-prolyl cis-trans isomerase	0.20	0.08
I4WAU6	3-dehydroquinate dehydratase	-	0.34
**I4WAZ4**	methylcrotonoyl-CoA carboxylase	0.03	-
**I4WB23**	4-hydroxy-tetrahydrodipicolinate synthase	0.08	0.13
**I4WB48**	methionine aminotransferase	0.04	0.40
I4WBE2	peptidase M28	0.04	-
I4WBP2	glycyl aminopeptidase	0.07	0.06
**I4WBY8**	arginine decarboxylase	0.03	0.04
**I4WBY9**	polyamine aminopropyl transferase	0.24	0.48
I4WC22	glutamine synthetase	0.13	-
**I4WC30**	Zn-dependent oligopeptidase	0.11	0.14
**I4WC93**	shikimate dehydrogenase	-	0.33
**I4WCE3**	branched-chain amino acid aminotransferase	-	0.09
I4WCR0	aminopeptidase N	0.10	-
**I4WCU6**	aromatic amino acid aminotransferase	0.34	0.58
I4WD72	peptidase M28	0.12	0.08
I4WD80	protease	0.17	0.63
I4WDN0	alanine dehydrogenase	-	0.07
**I4WDQ1**	leucine dehydrogenase	0.36	0.24
**I4WDT5**	ArgD, acetylornithine aminotransferase	0.22	0.40
I4WE81	arginase	0.03	-
**I4WEE3**	cysteine desulfurase	0.04	0.10

**I4WEG3**	ornithine/acetylornithine aminotransferase	0.10	0.45
**I4WEG6**	proline iminopeptidase	-	0.13
**I4WEG8**	D-3-phosphoglycerate dehydrogenase	0.43	0.16
I4WEP6	dipeptidyl aminopeptidase/acylaminoacyl peptidase	-	0.03
I4WEZ1	nitrogen regulatory protein P-II	0.50	0.38
I4WFS5	protease	0.11	-
**I4WFS8**	leucine aminopeptidase	0.04	0.03
I4WG51	cystathionine beta-lyase/cystathionine gamma-synthase	0.10	0.10
I4WG78	GcvT, aminomethyltransferase	0.40	0.14
**I4WG79**	glycine cleavage system H protein	0.86	0.46
**I4WGE0**	maleylacetoacetate isomerase	-	0.08
**I4WGE5**	cysteine synthase	0.17	0.46
I4WGQ3	peptidase M28	-	0.09
I4WH07	peptidase S10 serine carboxypeptidase	-	0.02
I4WH24	peptidyl-dipeptidase	0.03	0.21
**I4WHD2**	bifunctional proline dehydrogenase/pyrroline-5-carboxylate dehydrogenase	0.14	0.11
I4WHD9	peptidyl-dipeptidase	0.07	0.52
**I4WI88**	4-hydroxy-tetrahydrodipicolinate reductase	0.08	0.04
**I4WI91**	carbamoyl-phosphate synthase	0.08	-
**I4WI92**	carbamoyl phosphate synthase	0.08	-
I4WIP0	3-phosphoshikimate 1-carboxyvinyltransferase	0.11	0.04
**I4WIP2**	phosphoserine aminotransferase	0.10	0.12
**I4WIR5**	glycine dehydrogenase	0.08	0.15
I4WIT5	D-amino acid dehydrogenase	0.07	0.14
I4WIU0	HflC	-	0.23
**I4WIU1**	protease	0.17	0.21
I4WIU5	peptidase	0.15	0.15
I4WIU6	peptidase S46	-	0.03
**I4WIU8**	L-threonine 3-dehydrogenase	0.09	-
**I4WIV0**	2-amino-3-ketobutyrate coenzyme A ligase	0.13	0.23
**I4WIW2**	aspartate aminotransferase	0.24	1.49
**I4WIW4**	peptidyl-prolyl cis-trans isomerase	-	0.17
**I4WJA2**	serine hydroxymethyltransferase	0.12	0.38
I4WJL2	protease	0.06	0.07
I4WJL5	X-Pro dipeptidyl-peptidase	-	0.03
I4WJU9	ProC, pyrroline-5-carboxylate reductase	0.18	-
I4WKD9	methylthioribose-1-phosphate isomerase	-	0.03
**I4WKJ1**	histidine ammonia-lyase	0.04	-
**I4WL26**	protease	0.20	0.16
**I4WLF3**	tryptophan synthase beta chain	0.01	-
**I4WLH1**	imidazoleglycerol-phosphate dehydratase	-	0.03
**I4WLV4**	aminotransferase	0.11	0.34

**I4WM29**	acetylornithine deacetylase	0.06	0.04
I4WM35	metalloendopeptidase	0.47	0.76
I4WM64	prepilin-type N-terminal cleavage	0.14	-
**I4WM81**	NAD-glutamate dehydrogenase	0.08	0.10
**I4WM86**	glutaryl-CoA dehydrogenase	0.27	0.36
I4WMC6	peptidyl-prolyl cis-trans isomerase	0.06	-
**I4WMK5**	kynureninase	-	0.15
**I4WMM4**	carbamoyl phosphate synthase	0.03	-
**I4WN39**	Xaa-Pro dipeptidase	0.05	0.22
I4WN55	peptidase S8/S53	0.02	0.17
**I4WN66**	Indole-3-glycerol phosphate synthase	0.21	0.29
I4WN78	dipeptidase	0.03	-
I4WPB8	HutI, imidazolonepropionase	0.06	0.03
**I4WPC0**	N-formimino-L-glutamate deiminase	-	0.03
**I4WPD1**	urocanate hydratase	0.06	0.31
I4WPH1	Xaa-Pro aminopeptidase	-	0.02
I4WPJ5	peptidyl-prolyl cis-trans isomerase	0.18	0.20
I4WPK6	peptidyl-prolyl cis-trans isomerase	0.85	0.44
I4WQ39	argininosuccinate synthase	0.17	0.10
**I4WQ41**	acetylornithine deacetylase	0.03	-
**I4WQ42**	acetylglutamate kinase	-	0.04
**I4WQ44**	argininosuccinate lyase	0.11	-
I4WQ91	metallopeptidase	0.02	0.23
**I4WQ99**	peptidyl-prolyl cis-trans isomerase	0.26	-
I4WQQ8	gamma-glutamyltranspeptidase	-	0.07
I4WRE3	TrpA, tryptophan synthase	0.17	-
**I4WRG1**	histidinol dehydrogenase	0.05	-
**I4WRG9**	enolase-phosphatase	0.10	0.98
I4WRS1	aminopeptidase	0.14	0.03
I4WRX3	gamma-glutamyltranspeptidase	0.08	-
**I4WSM1**	aminopeptidase	-	0.11
**I4WSM4**	threonine aldolase	-	0.25
**I4WSY4**	branched-chain amino acid aminotransferase	0.23	0.91
**I4WSY6**	methylenetetrahydrofolate reductase	-	0.08
I4WT46	peptidase M28	0.28	0.22
I4WTB2	metallopeptidase	0.05	0.07
**I4WU28**	2,3,4,5-tetrahydropyridine-2,6-dicarboxylate N-succinyltransferase	0.31	0.45
**I4WVA0**	peptidyl-prolyl cis-trans isomerase	0.36	-
I4WZ73	X-Pro dipeptidyl-peptidase	0.04	-
M4NC29	Zn-dependent peptidase	-	0.02
**M4NEI4**	ornithine carbamoyltransferase	-	0.14

Carbohydrate metabolism

**I4VKM3**	inositol monophosphatase/fructose-1,6-bisphosphatase family protein	-	0.05
**I4WAH6**	phosphoglucomutase	0.08	0.10
**I4WAH7**	glucose-6-phosphate isomerase	0.04	0.16
**I4WAH8**	transaldolase	0.12	0.63
**I4WBW6**	ribokinase	-	0.18
**I4WBX9**	phosphomannomutase	0.02	0.09
**I4WCH0**	GDP-L-fucose synthase	0.07	-
I4WCI9	Fpb, fructose-1,6-bisphosphatase	0.09	0.11
I4WCL1	L-sorbosone dehydrogenase	0.08	0.13
**I4WCP3**	Glk PE, glucokinase	0.03	-
**I4WDU0**	6-phosphofructokinase	-	0.03
**I4WEL7**	2-dehydro-3-deoxy-6-phosphogalactonate aldolase	-	0.11
I4WEN5	6-phosphogluconate dehydrogenase-like protein	-	0.20
**I4WEQ2**	Entner-Doudoroff aldolase	0.45	0.75
**I4WEQ3**	phosphogluconate dehydratase	0.10	0.24
**I4WEQ4**	6-phosphogluconolactonase	0.44	0.42
**I4WEQ5**	glucose-6-phosphate 1-dehydrogenase	0.30	0.92
**I4WF95**	2,3-bisphosphoglycerate-dependent phosphoglycerate mutase	0.25	0.09
**I4WGD0**	glyceraldehyde-3-phosphate dehydrogenase	0.42	-
**I4WGD2**	Pyruvate kinase	0.48	0.62
**I4WGD4**	fructose-bisphosphate aldolase	0.33	0.49
I4WGD7	transketolase	0.11	0.75
**I4WGD9**	Pgk, phosphoglycerate kinase	0.13	0.21
I4WGM3	xylanase deacetylase	0.42	0.82
I4WH34	alpha,alpha-trehalase	0.05	-
**I4WHZ0**	phosphoketolase	-	0.06
I4WI35	propanediol utilization protein	0.29	-
I4WIU7	glycogen debranching enzyme	0.20	0.11
**I4WM90**	transketolase domain-containing protein	0.02	-
I4WN50	ribose-5-phosphate isomerase	0.35	0.52
I4WN86	glycoside hydrolase clan GH-D	-	0.01
**I4WN98**	ribulose-phosphate 3-epimerase	0.13	0.15
I4WPH4	enolase	0.33	1.17
I4WPK2	phosphoenolpyruvate synthase	0.13	0.08
**I4WQC6**	N-ethylammeline chlorohydrolase	0.09	0.25
**I4WRP0**	fructose-1,6-bisphosphatase	0.09	-
I4WRR0	cyclomaltodextrin glucanotransferase	0.20	0.38
**I4WRR9**	alpha-glucosidase	0.08	0.08
I4WU59	PEP phosphonomutase-like protein	-	0.07
**M4NMT8**	transaldolase	0.06	0.15
**Cell wall, membrane and envelope biogenesis**			
**I4VNC9**	glycosyl transferase family protein	-	0.09

**I4VVG1**	D-alanine--D-alanine ligase	0.08	-
I4VX44	LysM domain-containing protein	0.18	-
I4VZT9	C-terminal processing peptidase	0.05	-
**I4W030**	lipoprotein releasing system transmembrane protein LolC/E family	-	0.03
I4W2A0	pilus assembly protein	0.30	0.35
I4W2P0	membrane fusion protein	0.09	-
I4W6X0	UTP-glucose-1-phosphate uridylyltransferase	0.14	-
**I4WAP1**	BamD, outer membrane protein assembly factor	0.29	0.14
I4WAZ3	outer membrane protein	1.31	0.31
I4WB89	type 4 fimbrial biogenesis protein pily1-related protein	0.03	0.13
**I4WC49**	glycosyl transferase family protein	0.22	-
**I4WCG1**	glycosyl transferase family protein	0.06	0.05
**I4WCH4**	Class II pilin PilE	3.60	3.90
I4WDT8	UDP-N-acetylmuramate--L-alanine ligase	0.06	-
I4WDV8	glycosyltransferase	-	0.04
I4WEJ0	BamA .outer membrane protein assembly factor	0.09	0.23
**I4WEJ4**	lipid-A-disaccharide synthase	0.03	-
**I4WEQ7**	glucosamine-fructose-6-phosphate aminotransferase	0.11	-
I4WER7	lipid A 3-O-deacylase	0.19	-
I4WF26	Rhs family protein	-	0.01
I4WFA3	membrane fusion protein	-	0.08
I4WFN8	GlmU, bifunctional acetyl transferase/uridyl transferase	0.14	-
I4WFT7	D-alanyl-D-alanine carboxypeptidase	0.04	0.08
**I4WG42**	glucosamine--fructose-6-phosphate aminotransferase	-	0.13
I4WG67	aminotransferase	0.18	0.24
I4WGE3	OmpA/MotB domain-containing protein	-	0.07
**I4WGE6**	glucosamine--fructose-6-phosphate aminotransferase	0.15	-
I4WGM9	LysM domain-containing protein	0.79	0.51
**I4WGP0**	glycosyl/glycerophosphate transferase	-	0.09
I4WGU3	peptidoglycan-associated lipoprotein	3.39	6.24
I4WGU4	Tol-pal system protein	0.20	-
I4WH26	hemaglutitin	0.08	0.13
I4WI78	BamE, outer membrane protein assembly factor	-	0.15
I4WIC8	type IV pilus biogenesis/stability protein	0.05	0.12
I4WID1	BamB, outer membrane protein assembly factor	0.31	0.29
**I4WIN9**	3-deoxy-manno-octulosonate cytidylyltransferase	0.07	-
I4WIT4	alanine racemase	0.05	0.09
I4WIV2	peptidoglycan-associated protein	2.18	2.38
**I4WJ11**	capsular polysaccharide biosynthesis protein	0.04	0.04
I4WJS2	filamentous hemaglutin-like	0.77	0.29
**I4WJT4**	two-component system sensor histidine kinase-response regulator hybrid protein pill	0.05	0.05

I4WJT5	pilus biogenesis protein	0.22	0.17
**I4WM62**	Tfp pilus assembly protein FimT-like protein	-	0.08
I4WM66	PilY1, type 4 fimbrial biogenesis protein	0.03	0.03
**I4WM67**	PilE	-	0.09
I4WMA3	OmpA-like	-	0.54
**I4WNG6**	fimbrial assembly membrane protein	0.65	0.54
**I4WNG7**	fimbrial assembly membrane protein	0.29	0.40
**I4WNG8**	fimbrial assembly membrane protein	0.34	0.22
**I4WNG9**	penicillin-binding protein	-	0.02
I4WNH8	surface lipoprotein	0.03	0.13
I4WPA0	outer membrane lipoprotein	1.55	0.93
I4WPA1	outer membrane protein	1.06	0.24
**I4WPB6**	MsbA, lipid A export ATP-binding/permease protein	0.02	0.03
**I4WPH3**	2-dehydro-3-deoxyphosphooctonate aldolase	0.25	0.28
**I4WPK7**	UDP-glucose dehydrogenase	0.03	0.15
I4WQ35	outer membrane lipoprotein	1.21	0.61
I4WRC8	lytic murein transglycosylase	-	0.02
I4WRD8	FimV N-terminal domain-containing protein	0.43	0.13
**I4WRZ3**	flagellin domain-containing protein	-	0.29
I4WSL0	LPS-assembly protein LptD	0.08	0.05
**I4WT16**	UDP-N-acetylglucosamine--N-acetylmuramyl-(pentapeptide) pyrophosphoryl-undecaprenol N-acetylglucosamine transferase	0.12	0.05
I4WTB1	C-terminal processing peptidase	-	0.01
I4WUZ6	lytic murein transglycosylase	0.03	-
**Energy production and conversion**			
**I4VKE0**	isocitrate dehydrogenase	0.10	-
**I4VM16**	alcohol dehydrogenase	-	0.09
**I4VXG5**	succinyl-CoA ligase [ADP-forming] subunit alpha	0.28	-
**I4W1D3**	glyceraldehyde-3-phosphate dehydrogenase	-	0.19
**I4W1F4**	NADH:flavin oxidoreductase/NADH oxidase	-	0.02
**I4W1R0**	bifunctional malic enzyme oxidoreductase/phosphotransacetylase	-	0.02
I4W332	cytochrome c4	0.29	0.29
I4W6A9	high potential iron-sulfur protein	-	0.09
**I4W788**	isocitrate lyase	-	0.35
**I4W7P8**	rubrerythrin	-	0.33
**I4W977**	NADPH:quinone oxidoreductase	0.27	0.19
**I4W9P0**	fumarate hydratase class II	0.12	0.03
I4WAP4	succinyl-CoA ligase [ADP-forming] subunit beta	0.07	0.17
I4WB45	short-chain dehydrogenase	0.04	-
**I4WB57**	aconitate hydratase	0.22	0.61
I4WBN1	ubiquinol-cytochrome c reductase iron-sulfur subunit	0.52	0.34
**I4WBN2**	cytochrome b	-	0.04
I4WBN3	ubiquinol-cytochrome c reductase	0.39	0.26

I4WBP6	sulfur oxidation protein, flavocytochrome C	0.03	0.06
I4WBR2	DsrE/DsrF-like family protein	0.40	0.24
**I4WD44**	aldehyde dehydrogenase	-	0.12
**I4WD61**	oxydoreductase	0.08	-
**I4WDW2**	NADH dehydrogenase/NAD(P)H nitroreductase	0.21	0.12
**I4WDY5**	dihydrolipoamide dehydrogenase	0.38	0.13
**I4WDY6**	dihydrolipoamide acetyltransferase	0.78	0.24
**I4WE11**	pyruvate dehydrogenase E1 component	0.39	0.30
**I4WE12**	bifunctional malic enzyme oxidoreductase/phosphotransacetylase	0.15	-
**I4WE63**	aconitate hydratase	-	0.04
**I4WE70**	pyruvate dehydrogenase E1 alpha subunit	0.09	1.25
**I4WE71**	pyruvate/2-oxoglutarate dehydrogenase complex, dehydrogenase component beta subunit	0.46	1.82
I4WEG7	FAD/FMN-dependent dehydrogenase	0.19	0.68
I4WEU1	sulfide-quinone reductase	0.45	0.41
**I4WFA2**	cytochrome c	0.53	0.22
**I4WFB8**	isocitrate dehydrogenase	0.34	1.24
I4WFD5	NADPH:quinone reductase	0.18	-
**I4WFN1**	ATP synthase subunit b	0.62	0.35
I4WFN2	AtpH, ATP synthase subunit delta	0.18	-
**I4WFN3**	ATP synthase subunit alpha	0.54	0.66
I4WFN4	ATP synthase gamma chain	0.15	0.13
**I4WFN5**	ATP synthase subunit beta	0.56	0.55
I4WG68	electron transfer flavoprotein alpha subunit	0.36	0.69
**I4WG69**	electron transfer flavoprotein subunit beta	0.65	0.44
I4WG74	electron transfer flavoprotein-ubiquinone oxidoreductase	0.05	0.03
**I4WG85**	glucose-methanol-choline oxidoreductase	0.09	-
I4WGC7	azurin	0.75	0.34
**I4WH70**	NAD(P) transhydrogenase	0.26	0.30
**I4WH92**	FAD-dependent pyridine nucleotide-disulfide oxidoreductase	0.34	0.83
I4WHD0	cytochrome c oxidase subunit 2	0.05	0.05
**I4WHD3**	aldehyde dehydrogenase	0.15	0.07
I4WI10	short chain dehydrogenase/reductase f	0.09	0.07
**I4WIV5**	citrate synthase	0.29	-
**I4WIW1**	malate dehydrogenase	0.30	0.56
I4WJ90	voltage-gated potassium channel beta subunit	-	0.11
**I4WJ91**	alcohol dehydrogenase superfamily protein	0.16	0.26
**I4WJC7**	rubrerythrin	0.83	-
**I4WKC6**	2-oxoglutarate dehydrogenase	0.15	0.22
**I4WKC7**	2-oxoglutarate dehydrogenase	0.43	0.34
**I4WKC8**	dihydrolipoyl dehydrogenase	0.54	0.33
**I4WKE9**	NADH-quinone oxidoreductase subunit N	-	0.05
**I4WKF6**	NADH dehydrogenase subunit G	0.08	0.04

**I4WKF7**	NADH-quinone oxidoreductase	0.07	-
**I4WKG0**	NADH-quinone oxidoreductase	0.13	-
**I4WLR8**	NADH-quinone oxidoreductase subunit C	-	0.15
I4WMA7	cytochrome c family protein	0.05	0.08
**I4WML0**	short-chain dehydrogenase	0.19	0.19
**I4WNH1**	citrate synthase	0.48	0.45
**I4WP39**	cytochrome c biogenesis protein	0.05	-
I4WPA7	succinate dehydrogenase	0.09	0.05
**I4WPA8**	succinate dehydrogenase	0.14	0.17
I4WQ25	FAD linked oxidase domain-containing protein	-	0.07
I4WRH2	high potential iron-sulfur protein	0.33	-
**I4WSC0**	NADP oxidoreductase	0.14	0.21
**I4WSY9**	phosphoenolpyruvate carboxylase	0.18	0.12
I4WU19	short-chain dehydrogenase	0.12	0.37
I4WU37	nitrous-oxide reductase	-	0.08
**I4WUC8**	NADPH dehydrogenase	0.11	0.24
**I4WW98**	pyruvate dehydrogenase	0.29	-
**M4NS93**	citrate synthase	-	0.47
**M4NTL5**	ATP synthase subunit delta	-	0.03
**Lipid metabolism and ketogenesis**			
**I4VIC2**	acyl carrier protein	-	1.44
**I4VRU7**	3-hydroxyacyl-CoA dehydrogenase	-	0.28
**I4VV45**	YdiF, acetate-CoA transferase	-	0.13
**I4VY74**	3-oxoacyl-ACP reductase	-	0.07
I4VZY3	acetoacetyl-CoA reductase	-	0.06
**I4W091**	acyl-CoA dehydrogenase domain-containing protein	0.04	-
I4WAL3	3-hydroxydecanoyl-[acyl-carrier-protein] dehydratase	-	0.08
I4WAL4	beta-ketoacyl-[ACP] synthase I	0.12	0.43
I4WAZ2	acetoacetyl-CoA thiolase	0.66	0.66
**I4WB09**	3-hydroxybutyryl-CoA dehydratase	0.13	-
**I4WB55**	long-chain fatty acyl-CoA ligase	-	0.07
**I4WBU8**	acetyl-coenzyme A synthetase	0.11	0.12
I4WBY7	3-hydroxybutyrate dehydrogenase	-	0.09
I4WBZ6	acetyl-CoA acetyltransferase	0.13	0.07
I4WCN8	GpsA, glycerol-3-phosphate dehydrogenase	0.13	0.07
**I4WDA0**	acyl-CoA dehydrogenase	0.04	0.25
**I4WE25**	fatty acid binding protein	0.15	0.09
**I4WE72**	branched-chain alpha-keto acid dehydrogenase subunit E2	0.38	0.22
**I4WEJ2**	FabZ, 3-hydroxyacyl-[acyl-carrier-protein] dehydratase	-	0.29
**I4WEK0**	acetyl-coenzyme A carboxylase carboxyl transferase	-	0.04
I4WFP4	fatty acyl-CoA synthetase	0.16	0.05
I4WGM2	lipase	0.14	0.09

**I4WIC3**	acetyl-CoA acetyltransferase	0.23	0.62
**I4WIC4**	3-hydroxyacyl-CoA dehydrogenase	0.20	0.94
**I4WJC3**	polyhydroxyalkanoate depolymerase	0.07	-
**I4WKE0**	poly(R)-hydroxyalkanoic acid synthase, class III, PhaC subunit	0.14	0.05
I4WKE1	poly(R)-hydroxyalkanoic acid synthase subunit	0.05	-
**I4WKH0**	3-oxoacyl-[acyl-carrier-protein] synthase	0.12	0.21
**I4WKH1**	acyl carrier protein	0.11	-
I4WKH2	3-ketoacyl-[acyl-carrier-protein] reductase	0.20	0.04
I4WKH3	malonyl-CoA-acyl carrier protein transacylase	0.14	0.31
**I4WKH4**	3-oxoacyl-[acyl-carrier-protein] synthase	0.16	0.13
**I4WKP8**	acyl-CoA dehydrogenase	-	0.04
**I4WM78**	acyl-CoA dehydrogenase domain-containing protein	-	0.12
I4WM94	acyl-CoA dehydrogenase	-	0.06
**I4WM95**	acyl-CoA dehydrogenase	-	0.03
I4WM96	acetoacetate decarboxylase	0.20	0.13
**I4WMY1**	phasin	1.86	5.43
**I4WP46**	trans-2-enoyl-CoA reductase	0.11	0.25
**I4WPL0**	enoyl-CoA hydratase	0.19	0.20
I4WPU1	acyl-CoA dehydrogenase	-	0.05
**I4WR42**	acyl carrier protein	0.10	0.35
**I4WR77**	enoyl-CoA hydratase	0.08	-
I4WSC3	lipase	0.08	0.20
**I4WSG8**	acyl-CoA dehydrogenase	0.37	1.02
**I4WU86**	acyl-CoA thiolesterase	-	0.16
I4WUC2	lipase	-	0.07
I4WUD4	acetoacetyl-CoA reductase	0.17	-
**I4WZ09**	YdiF, acetate CoA-transferase	0.13	-
**M4NHA9**	acyl-CoA dehydrogenase	-	0.36
**Replication, transcription, translation and DNA repair**			
**G4FZ48**	elongation factor Tu	1.83	1.03
**I4VIS9**	NusG, transcription termination/antitermination protein	-	0.10
**I4VIT0**	50S ribosomal protein L11	0.43	0.43
**I4VIT2**	50S ribosomal protein L10	0.42	-
I4VIT3	50S ribosomal protein L7/L12	-	0.08
**I4VK78**	methionine--tRNA ligase	0.04	-
**I4VLN4**	RNA polymerase sigma factor RpoD	0.04	0.03
**I4VM70**	30S ribosomal protein S10	0.42	0.25
**I4VM75**	30S ribosomal protein S19	-	0.30
I4VM80	30S ribosomal protein S17	0.45	-
**I4VM82**	50S ribosomal protein L24	0.59	0.50
I4VM90	50S ribosomal protein L15	-	0.13
**I4VM95**	DNA-directed RNA polymerase subunit alpha	-	0.42

**I4VN96**	30S ribosomal protein S6	-	0.22
**I4VQ72**	30S ribosomal protein S21	0.39	-
I4VS93	50S ribosomal protein L7/L12	0.31	-
**I4VWM0**	chromosome partition protein	-	0.01
**I4VXT9**	DNA topoisomerase 1	-	0.03
I4VZ43	30S ribosomal protein S11	0.64	0.14
**I4VZ44**	30S ribosomal protein S13	-	0.38
I4VZ46	50S ribosomal protein L15	0.37	-
**I4VZ51**	30S ribosomal protein S8	1.18	-
I4VZ55	50S ribosomal protein L14	0.50	0.77
I4VZ56	30S ribosomal protein S17	-	0.30
I4VZ58	50S ribosomal protein L16	0.17	-
**I4VZ59**	30S ribosomal protein S3	0.13	0.25
**I4VZC9**	30S ribosomal protein S2	-	0.40
**I4W0F8**	Crp/Fnr family transcriptional regulator	0.07	-
I4W1I1	amidase	0.09	-
I4W447	RecA	0.24	0.11
**I4W664**	N5-glutamine S-adenosyl-L-methionine-dependent methyltransferase	0.04	-
**I4W6N1**	nucleoid DNA-binding protein	7.00	2.85
**I4WA42**	serine--tRNA ligase	0.07	0.17
**I4WAQ1**	Fis family transcriptional regulator	0.18	0.05
**I4WB47**	transcriptional regulator	-	0.10
**I4WBV8**	DNA gyrase subunit B	0.03	0.03
**I4WBW0**	DNA polymerase III subunit beta	0.66	0.32
**I4WBX1**	tyrosine--tRNA ligase	0.09	0.04
I4WBY3	ArgS, arginine--tRNA ligase	0.02	-
**I4WC48**	chromosome partitioning protein	0.08	-
**I4WC56**	transcriptional regulator	0.12	0.08
**I4WCN6**	50S ribosomal protein L28	0.28	-
I4WCP9	Rho, transcription termination factor	0.17	0.11
**I4WCU7**	RdgC, recombination-associated protein	0.04	-
**I4WD05**	RhlB, ATP-dependent RNA helicase	0.02	-
**I4WDR8**	50S ribosomal protein L23	-	0.23
**I4WEH2**	phenylalanine--tRNA ligase beta subunit	0.08	-
**I4WEH6**	translation initiation factor IF-3	0.31	-
**I4WEI2**	30S ribosomal protein S2	0.66	-
**I4WEI3**	elongation factor Ts	0.24	0.15
**I4WF04**	NADPH-dependent 7-cyano-7-deazaguanine reductase	-	0.17
**I4WFK0**	Glycine--tRNA ligase beta subunit	0.04	0.02
I4WG27	50S ribosomal protein L25	0.15	-
**I4WGB3**	50S ribosomal protein L1	0.48	0.27
**I4WGB4**	50S ribosomal protein L10	0.34	0.26

**I4WGF0**	Mg-dependent DNase	0.07	0.25
**I4WGM7**	DNA topoisomerase 1	0.06	-
**I4WGT1**	aspartate--tRNA ligase	0.16	0.12
**I4WHZ5**	GreA/GreB family elongation factor	0.20	0.20
**I4WI04**	exodeoxyribonuclease I	-	0.03
**I4WI69**	ribonuclease E	0.12	0.04
**I4WI93**	GreA, transcription elongation factor	0.29	0.24
**I4WI99**	30S ribosomal protein S1	0.46	0.12
**I4WIK9**	lysine--tRNA ligase	0.10	-
**I4WIR1**	ribonuclease R	0.01	-
**I4WIT6**	regulatory protein	-	0.05
**I4WIU3**	Hfq, RNA-binding protein	0.41	-
**I4WJD2**	glutamine--tRNA ligase	0.04	-
I4WJK2	ValS, valine-tRNA ligase	0.06	-
**I4WJK8**	proline--tRNA ligase	0.06	0.16
**I4WJK9**	DNA-binding protein H-NS	3.21	0.71
**I4WKB9**	transcriptional regulator	0.73	0.27
**I4WKC4**	transcription factor	0.07	0.04
**I4WKD8**	DNA gyrase	0.05	0.03
I4WKE2	polyribonucleotide nucleotidyltransferase	0.45	1.07
**I4WKE6**	translation initiation factor IF-2	0.12	0.07
**I4WKE7**	NusA, transcription elongation factor	0.09	0.18
I4WKH5	50S ribosomal protein L32	0.43	-
**I4WKI6**	DNA polymerase III	0.02	0.06
**I4WKJ2**	histidine--tRNA ligase	-	0.05
**I4WKR3**	50S ribosomal protein L3	-	0.52
**I4WKR6**	50S ribosomal protein L2	-	0.20
**I4WKR7**	30S ribosomal protein S19	0.45	-
**I4WKT0**	30S ribosomal protein S5	0.58	0.31
I4WKT6	30S ribosomal protein S4	-	0.54
**I4WKT7**	DNA-directed RNA polymerase subunit alpha	0.92	-
I4WKT8	50S ribosomal protein L17	0.68	-
**I4WKU7**	ribonuclease G	0.02	-
**I4WL10**	elongation factor G	0.25	0.22
**I4WL11**	30S ribosomal protein S7	1.41	0.56
**I4WL13**	DNA-directed RNA polymerase subunit beta	0.40	0.18
**I4WL14**	DNA-directed RNA polymerase subunit beta	0.17	0.14
**I4WL19**	NusG, transcription termination/antitermination protein	0.52	-
I4WL23	elongation factor Tu	-	0.91
I4WL41	amidase	0.04	0.02
I4WL46	30S ribosomal protein S4	0.31	-
**I4WL48**	30S ribosomal protein S13	0.88	-

I4WL53	50S ribosomal protein L18	0.79	0.58
**I4WL54**	50S ribosomal protein L6	0.22	-
**I4WL55**	30S ribosomal protein S8	-	0.75
**I4WL57**	50S ribosomal protein L5	0.45	0.95
I4WL64	50S ribosomal protein L22	0.33	0.17
**I4WL66**	50S ribosomal protein L2	0.35	-
**I4WL67**	50S ribosomal protein L23	0.64	-
**I4WL68**	50S ribosomal protein L4	0.64	0.66
**I4WL69**	50S ribosomal protein L3	0.28	-
**I4WM58**	isoleucine--tRNA ligase	0.06	0.25
**I4WMK9**	elongation factor P	0.02	0.05
I4WN52	30S ribosomal protein S9	0.43	0.46
**I4WN53**	50S ribosomal protein L13	0.19	0.81
**I4WN63**	Crp, transcriptional regulator	0.11	-
**I4WNH2**	50S ribosomal protein L31 type B	0.15	-
**I4WNH5**	endoribonuclease	0.88	1.73
I4WNL3	50S ribosomal protein L17	-	0.53
**I4WNP6**	ribosome-recycling factor	-	0.02
**I4WP50**	MarR family transcriptional regulator	-	0.06
**I4WP94**	polyhydroxyalkonate synthesis repressor	0.21	-
**I4WPI7**	RNA-binding protein	0.23	-
I4WPL6	LepA, elongation factor	0.03	-
**I4WPU8**	single-stranded DNA-binding protein	0.19	1.42
**I4WPX4**	FusA, elongation factor G	-	0.07
**I4WQ37**	transcriptional regulator	-	0.07
**I4WQA4**	endoribonuclease	-	0.07
**I4WQB0**	asparagine--tRNA ligase	0.13	0.13
**I4WQB2**	30S ribosomal protein S6	0.56	-
I4WQB3	30S ribosomal protein S18	0.61	-
**I4WQB4**	50S ribosomal protein L9	1.16	0.56
**I4WQB9**	30S ribosomal protein S16	0.67	-
**I4WQC2**	50S ribosomal protein L19	0.75	0.32
**I4WQD3**	DksA, RNA polymerase-binding transcription factor	0.13	-
**I4WQM0**	leucine--tRNA ligase	0.05	0.05
**I4WRF0**	glutamate--tRNA ligase	0.16	0.05
**I4WT09**	ribosomal RNA small subunit methyltransferase	0.15	-
**I4WT42**	MarR family transcriptional regulator	0.32	0.06
**I4WTM6**	cysteine--tRNA ligase	0.04	-
**I4WTZ3**	Crp/Fnr family transcriptional regulator	-	0.04
I4WU00	metallo-beta-lactamase family protein, RNA-specific	0.02	0.04
I4WU53	regulatory protein	-	0.07
I4WUG6	alanine--tRNA ligase	0.07	0.04

**I4WYD0**	poly(A) polymerase I	-	0.10
M4NEI0	DksA, RNA polymerase-binding protein	0.13	0.02
**Stress and defense mechanisms**			
I4VQY6	hemolysin D	-	0.02
I4VYT9	anaerobically induced outer membrane transmembrane protein	-	0.38
**I4VZV3**	thioredoxin	-	0.42
I4W2P1	ariflavin resistance protein D	-	0.02
**I4W6N0**	Lon protease	0.02	-
I4W949	cold shock protein	1.63	1.25
**I4WAB5**	AhpD, alkyl hydroperoxide reductaseS	0.04	0.54
**I4WAV1**	10 kDa chaperonin	3.60	16.30
**I4WAV2**	60 kDa chaperonin	3.14	2.01
**I4WB26**	PhoH family protein, phosphate starvation inducible protein	0.11	0.07
**I4WB63**	GTP-binding protein TypA/BipA	0.04	-
**I4WBN4**	stringent starvation protein	0.17	-
**I4WBS7**	ATP-dependent protease ATPase subunit HslU	0.46	0.26
I4WC57	salt-induced outer membrane protein	0.38	0.48
**I4WDA1**	OsmC-like protein	-	0.07
I4WDM9	thioredoxin reductase	0.13	0.07
I4WE54	anaerobically induced outer membrane protein	0.63	1.69
**I4WEF2**	aminoglycoside phosphotransferase	-	0.01
I4WEG4	PsiF repeat family protein	0.33	-
**I4WEU5**	acriflavin resistance protein	0.03	0.05
**I4WEZ6**	CRISPR-associated protein Csd2 family	-	0.06
**I4WFB4**	CRISPR-associated protein	-	0.12
I4WG36	thiol-disulfide isomerase-like thioredoxin	0.24	0.18
**I4WG89**	chaperonin	-	0.08
**I4WGC1**	anaerobically induced outer membrane protein	0.04	0.52
I4WGS4	chaperone	0.12	0.06
**I4WI84**	GrpE, chaperone	0.21	0.79
**I4WI86**	DnaK, chaperone	0.54	0.88
I4WIA8	beta-lactamase	0.11	-
I4WIQ0	HflD, high frequency lysogenization protein	0.11	-
**I4WIV8**	alkyl hydroperoxide reductase	1.55	2.60
**I4WJB1**	carbon starvation protein	-	0.02
**I4WJR0**	superoxide dismutase	0.76	5.60
I4WKI2	starvation-inducible outer membrane lipoprotein	1.05	1.02
I4WMA1	starvation-inducible lipoprotein	0.19	-
**I4WPF1**	activator of Hsp90 ATPase 1 family protein	-	0.44
**I4WQL5**	thioredoxin	0.19	0.27
**I4WRQ8**	AhpD, alkyl hydroperoxide reductase	0.23	-
I4WSK9	SurA, chaperone	0.41	1.25

**I4WUA1**	thioredoxin	0.22	0.13
I4WUC5	cold shock protein	0.38	-
I4WUC6	cold shock protein	2.69	-
**I4WUD7**	HtpG, chaperone protein	0.31	0.22
**I4WUF3**	catalase	0.31	0.82
**I4WYA6**	competence/damage-inducible protein CinA-like	-	0.12
**Transport, secretion and efflux**			
**I4VLQ8**	bacterioferritin	0.19	-
**I4VNB1**	YajC, preprotein translocase subunit	-	0.38
**I4VRS1**	sortase system response regulator	0.08	0.10
**I4VUA8**	ExbB, biopolymer transport protein	-	0.31
**I4VUA9**	TonB family protein	0.65	-
**I4VVG8**	translocase subunit SecA	0.05	0.03
**I4VYG3**	FtsY, signal recognition particle receptor	-	0.06
**I4VYN0**	TolQ protein	0.14	0.16
I4VYN5	YbgF, Tol-pal system protein	-	0.05
**I4W0B9**	FtsY, signal recognition particle receptor	0.07	-
I4W1W5	Oar protein	-	0.02
I4W345	outer membrane receptor protein	0.03	-
**I4W9D2**	twin arginine-targeting protein translocase	-	0.36
I4WAP8	TonB-dependent receptor	0.03	0.15
**I4WBA3**	bacterioferritin	-	0.36
**I4WBV4**	ExbD1, biopolymer transport protein	0.03	0.30
**I4WBV5**	ExbB, biopolymer transport protein	1.25	-
I4WBW4	YidC, insertase	0.04	-
I4WBX8	ABC transporter	0.19	-
I4WC41	TonB dependent receptor protein	-	0.02
I4WDN2	LolA, outer-membrane lipoprotein carrier protein	0.31	0.52
**I4WDU8**	bacterioferritin	0.14	-
I4WDV1	TolC family type I secretion outer membrane protein	0.63	0.81
I4WDW0	Na^+/proline symporter	0.06	0.05
I4WE23	TonB-dependent outer membrane receptor protein	0.67	1.61
**I4WEF4**	SecF, protein-export membrane protein	-	0.06
I4WEF5	SecD, translocase	0.04	0.09
I4WEQ9	TonB-dependent receptor	0.33	0.34
I4WEU4	RND family efflux transporter	0.29	0.18
I4WEX9	TonB-dependent receptor	0.35	0.33
I4WFK2	ferrienterochelin and colicin receptor	1.44	1.75
I4WFT1	cobalamin receptor protein	0.19	0.29
**I4WG81**	TonB protein	0.30	0.14
I4WGD3	TonB family protein	0.06	0.13
I4WGD5	OmpB-like	2.90	3.24

I4WGD6	autotransorter	-	0.13
I4WGS8	ferric enterobactin receptor	0.04	0.14
**I4WGU0**	TolR protein	-	0.09
I4WGU2	TolB	0.36	0.26
I4WH55	TonB-dependent siderophore receptor	0.11	0.17
**I4WH99**	sodium ABC transporter ATP-binding protein	-	0.07
**I4WHB0**	ABC transporter ATP-binding protein	-	0.06
I4WHD4	long chain fatty acid transport protein	0.12	0.25
I4WIQ1	porin	0.13	0.26
I4WIQ3	PstS, phosphate-binding protein	0.74	0.76
**I4WIQ6**	PstB, phosphate import ATP-binding protein	-	0,05
**I4WIQ7**	PhoU, phosphate-specific transport system accessory protein	0.54	-
I4WIX0	TonB-dependent receptor	0.07	0.04
I4WJ71	TonB-dependent receptor	0.14	0.20
**I4WJJ4**	preprotein translocase	0.21	-
I4WJQ5	TonB-dependent outer membrane receptor	0.13	0.24
I4WJU6	TonB dependent receptor protein	0.23	0.34
I4WL02	outer membrane efflux protein	-	0.04
I4WM79	hydrophobe/amphiphile efflux-1 (HAE1) family transporter	0.05	0.05
I4WM80	multidrug resistance protein	0.23	0.12
I4WNG4	type IV pilus secretin	0.34	0.05
I4WNI0	ABC transporter auxiliary protein	0.76	0.70
**I4WNI1**	ABC transporter periplasmic protein	0.10	0.05
**I4WNI3**	ABC transporter	0.03	0.12
I4WP57	ABC transporter permease	-	0.02
**I4WP96**	efflux pump membrane protein	0.05	0.05
**I4WPE6**	RNA signal recognition particle 4.5S RNA	-	0.19
**I4WPJ0**	trigger factor	-	0.05
I4WPL7	signal peptidase	-	0.05
I4WPX5	TonB-dependent receptor	0.03	0.04
I4WQ75	TonB-dependent receptor protein	0.08	0.08
**I4WR37**	hemolysin D	0.32	0.11
I4WRP8	sugar transporter	-	0.07
I4WRR8	outer membrane protein	0.20	0.26
**I4WSA6**	ferritin	0.50	0.21
**I4WSX0**	ABC-type transporter	0.11	0.08
I4WSX8	Fe^{2+}-dicitrate sensor	0.08	0.11
**I4WT49**	RND family efflux transporter	0.07	-
I4WTY3	TonB-dependent outer membrane receptor	0.12	0.07
I4WU14	efflux pump membrane fusion protein	0.09	0.04
I4WU70	polyketide cyclase/dehydrase and lipid transport	0.73	1.13
I4WUB7	metal ion ABC transporter substrate-binding protein	0.90	0.66
I4WUE7	TonB-dependent siderophore receptor	0.08	0.12

I4WWB0	outer membrane cobalamin receptor protein	0.33	-
**I4WZB9**	ABC transporter ATP-binding protein	0.09	-
**Other functions**			
I4VJ32	serine/threonine phosphatase with Cache sensor	-	0.01
I4VJL1	dTDP-glucose 4.6-dehydratase	-	0.05
I4VKW0	Cell division protein	0.10	-
**I4VP03**	MoxR-like ATPase	-	0.06
I4VPB8	modulator of FtsH protease	0.29	-
I4VPN3	cell division protein	-	0.04
I4VPP0	partition protein	-	0.11
**I4VQ36**	FtsE, cell division ATP-binding protein	0.04	-
**I4VQR5**	MreB, rod shape-determining protein	-	0.11
**I4VTQ2**	AMP nucleosidase	-	0.09
**I4VV38**	response regulator consisting of a CheY-like receiver domain and a Fis-type HTH domain protein	0.18	-
**I4VV66**	PilH, two-component system response regulatory protein	0.18	-
**I4VV67**	response regulator receiver protein	0.42	-
**I4VXH1**	two-component system response regulator protein	0.12	0.06
**I4VZJ1**	response regulator receiver domain-containing protein	-	0.27
**I4W0C6**	NTP pyrophosphohydrolase	-	0.07
**I4W2S6**	ribose-phosphate pyrophosphokinase	0.12	-
**I4W459**	sulfate adenylyltransferase subunit 2	-	0.04
**I4W782**	protein kinase	-	0.02
**I4W8Y5**	amidohydrolase	-	0.11
**I4W9N8**	glutathione S-transferase	-	0.02
**I4W9N9**	adenylosuccinate lyase	-	0.21
**I4W9T6**	methyltransferase	-	0.16
**I4WAH3**	HemC, porphobilinogen deaminase	0.16	-
I4WAT9	PurH, bifunctional purine biosynthesis protein	0.09	0.04
**I4WAZ7**	ObgE, GTPase	0.04	-
I4WB46	metal-dependent amidohydrolase	0.10	0.11
**I4WB49**	amidohydrolase	-	0.08
I4WBQ6	5'-nucleotidase domain-containing protein	0.03	-
**I4WBV2**	PdxJ, pyridoxine 5'-phosphate synthase	0.15	-
I4WBZ2	partition protein	0.17	-
I4WCC3	alkaline phosphatase	-	0.03
I4WCC6	5'-nucleotidase domain-containing protein	0.08	0.04
**I4WCF1**	thymidylate synthase	0.08	0.23
**I4WCF2**	dihydrofolate reductase	0.28	-
I4WCL9	Amidohydrolase	-	0.04
**I4WCM6**	thymidine kinase	-	0.09
**I4WCP1**	haloacid dehalogenase-like hydrolase	-	0.20

I4WCP5	delta-aminolevulinic acid dehydratase	0.06	0.49
**I4WCP7**	dihydroorotase	0.12	0.39
**I4WCP8**	glutamate--cysteine ligase	0.04	0.17
**I4WD04**	thioredoxin	0.17	-
I4WD67	methyltransferase	0.09	-
I4WD71	metallo carboxipeptidase	0.04	-
**I4WDN1**	FtsK, DNA segregation ATPase	-	0.02
**I4WDT2**	thiamine-phosphate synthase	0.11	-
I4WDT3	HemL, glutamate-1-semialdehyde 2.1-aminomutase	0.10	0.12
I4WDT9	Adk, adenylate kinase	1.53	1.18
**I4WDU5**	inorganic pyrophosphatase	0.08	0.50
**I4WDV0**	L-isoaspartate carboxylmethyltransferase	-	0.13
I4WDW1	lipid/polyisoprenoid binding protein	2.76	1.19
**I4WE19**	aspartyl/asparaginyl beta-hydroxylase	0.02	-
**I4WE39**	NAD-dependent epimerase/dehydratase	0.11	0.48
**I4WE90**	response regulator with CheY-like receiver domain	0.69	0.29
**I4WEG5**	uracil phosphoribosyltransferase	0.09	-
**I4WEI4**	uridylate kinase	0.08	0.12
**I4WF02**	glutathione S-transferase	0.21	-
**I4WF09**	MreB, rod shape-determining protein	0.25	-
**I4WF36**	UbiB, kinase	0.02	-
**I4WF93**	oxygen-dependent coproporphyrinogen-III oxidase	-	0.22
I4WF97	N-acyl-L-amino acid amidohydrolase	-	0.04
I4WFD4	amidohydrolase	0.02	0.02
I4WG63	acetyl transferase	-	0.03
I4WG75	amidase	-	0.03
**I4WG76**	sulfate adenylyltransferase	0.17	-
**I4WG77**	bifunctional sulfate adenylyltransferase /adenylylsulfate kinase protein	0.24	0.10
**I4WG87**	signal transduction protein	0.20	0.10
I4WGC2	formylglycine-generating sulfatase family protein	0.39	0.29
I4WGC3	SCO1 /SenC family protein	0.36	-
**I4WGC5**	thiol peroxidase	0.21	0.29
I4WGP6	glutamine cyclotransferase	0.14	0.24
**I4WGV1**	laccase-domain protein	0.36	-
**I4WH93**	RmuC domain-containing protein	0.05	0.03
**I4WH94**	GTP cyclohydrolase	0.33	0.04
I4WHH6	uroporphyrin-III C-methyltransferase	0.28	-
**I4WIB5**	pirin	0.10	0.34
**I4WIC6**	nucleoside diphosphate kinase	0.63	1.53
**I4WID0**	GTP binding protein	0.19	0.13
**I4WID4**	inosine-5'-monophosphate dehydrogenase	-	0.10
**I4WID5**	GMP synthase	0.05	-

**I4WIK5**	ZipA, cell division protein	0.08	-
**I4WIM0**	TraB, pheromone shutdown-related protein	0.03	-
I4WIN6	histone-like protein	3.65	1.50
**I4WIT8**	adenylosuccinate synthetase	0.24	0.21
**I4WIV3**	2-methylcitrate dehydratase	0.16	0.32
**I4WJ80**	acetylornithine aminotransferase	-	0.19
I4WJ85	Parathion hydrolase	0.03	-
**I4WJ96**	6,7-dimethyl-8-ribityllumazine synthase	0.22	-
**I4WJ97**	L.D-carboxypeptidase A	0.06	0.21
**I4WJ98**	3,4-dihydroxy-2-butanone 4-phosphate synthase	0.06	0.10
I4WJA5	orotidine 5'-phosphate decarboxylase	-	0.05
I4WJB8	phosphoribosylformylglycinamidine synthase	-	0.02
I4WJQ4	Rossmann fold nucleotide-binding protein	-	0.07
I4WJR1	N5-carboxyaminoimidazole ribonucleotide synthase	0.11	-
**I4WJR2**	N5-carboxyaminoimidazole ribonucleotide mutase	0.28	-
**I4WJS9**	adenosylmethionine-8-amino-7-oxononanoate aminotransferase	-	0.04
**I4WJT2**	chemotaxis-related protein	0.25	0.03
**I4WJT6**	chemotaxis signal transduction protein pili	-	0.10
**I4WJT9**	glutathione synthetase	0.08	-
**I4WJU3**	aspartate carbamoyltransferase	0.13	0.01
**I4WJU7**	twitching motility protein	-	0.02
**I4WJU8**	twitching motility protein	0.28	0.09
**I4WKD1**	response regulator receiver protein	0.54	0.12
**I4WKD2**	DnaJ-class molecular chaperone	0.11	-
**I4WKH9**	MoxR-like ATPase	0.04	-
**I4WKI3**	diadenosine tetraphosphate hydrolase	-	0.38
**I4WMK7**	2-amino-3-carboxymuconate-6-semialdehyde decarboxylase	-	0.05
**I4WNA1**	phosphoribosylaminoimidazole-succinocarboxamide synthase	0.14	0.03
**I4WNG3**	MoxR-like ATPase	0.11	-
I4WNH3	inosine-uridine nucleoside N-ribohydrolase	-	0.12
**I4WNV0**	sulfatase-modifying factor protein	0.05	-
I4WP91	2-polyprenyl-6-methoxyphenol hydroxylase-like oxidoreductase	0.04	-
**I4WPE1**	3-demethylubiquinone-9 3-methyltransferase	-	0.26
**I4WPF7**	glutathione S-transferase	-	0.10
I4WPG0	penicillin acylase	-	0.02
I4WPG1	succinate-CoA transferase	0.04	0.04
I4WPG2	3-demethylubiquinone-9 3-methyltransferase	-	0.33
I4WPH2	PyrG, CTP synthase	0.10	-
**I4WPJ1**	ATP-dependent Clp protease	0.24	0.87
I4WPM8	hypoxanthine-guanine phosphoribosyltransferase	0.12	0.10
**I4WPM9**	S-methyl-5'-thioinosine phosphorylase	-	0.11
**I4WPV1**	geranylgeranyl pyrophosphate synthase	0.06	0.15

I4WPV4	polyisoprenoid-binding protein	-	0.08
I4WPW4	polyisoprenoid-binding protein	0.05	0.05
**I4WQ95**	S-formylglutathione hydrolase	-	0.07
I4WQC3	PAS/PAC sensor-containing diguanylate cyclase/phosphodiesterase	0.07	0.07
**I4WQC8**	UbiG, ubiquinone biosynthesis O-methyltransferase	0.08	-
**I4WQL8**	PqiB family protein	0.05	0.03
**I4WRC2**	carboxymethylenebutenolidase	0.25	-
I4WRC7	ATPase	0.05	0.04
**I4WRK0**	bifunctional folylpolyglutamate synthase/dihydrofolate synthase	0.03	-
I4WRN4	isoprenoid biosynthesis protein	0.18	0.14
**I4WSD6**	nucleotide-binding protein	0.03	-
I4WSL8	GTP binding protein	0.04	-
**I4WSY2**	response regulator with CheY-like receiver domain	0.36	0.26
**I4WSY8**	adenosylhomocysteinase	-	0.03
**I4WSZ6**	S-adenosylmethionine synthase	0.14	0.28
I4WT06	LppC family lipoprotein	0.13	0.16
**I4WT08**	MraZ, transcriptional regulator	0.24	0.12
I4WU93	acetyl transferase	0.23	-
I4WUC9	pirin	0.23	0.11
**I4WV01**	phosphoglycerol transferase family protein, alkaline phosphatase superfamily	-	0.04
I4WV06	pteridine reductase	-	0.11
I4WVE3	disulfide isomerase	0.24	0.53
I4WVE4	thiol:disulfide interchange protein	0.17	0.19
I4WVX6	(p)ppGpp synthetase	-	0.01
**I4WXN8**	inosine-5'-monophosphate dehydrogenase	0.07	-
I4WYH0	N5-carboxyaminoimidazole ribonucleotide synthase	-	0.04
M4NDJ5	heme biosynthesis enzyme	-	0.05
**Function unknown**			
I4VQ20		-	0.19
I4VSJ4		0.04	-
I4VV34		-	0.35
**I4W9G3**		-	0.06
I4W9L9		0.21	-
I4WAA1		0.81	0.23
I4WB04		0.08	0.05
I4WB10		0.21	0.44
I4WB40		0.10	-
I4WBM4		-	0.02
**I4WBS3**		0.25	0.32
I4WBT2		0.06	0.06
I4WBV7		0.72	0.44

I4WC23	-	0.50
I4WC53	-	0.07
**I4WCF0**	0.09	-
I4WD84	0.11	0.13
**I4WDU6**	0.28	0.20
**I4WDZ3**	0.11	-
I4WDZ5	0.09	0.19
I4WE01	0.09	-
I4WE02	0.15	-
**I4WE09**	0.15	-
**I4WE16**	0.17	0.12
I4WEN9	-	0.14
**I4WEU3**	0.57	2.33
I4WEX6	0.01	0.01
I4WF92	-	0.09
I4WF94	0.45	0.02
**I4WFK9**	0.14	-
I4WFL9	0.02	0.01
**I4WFM7**	5.89	9.61
**I4WG57**	-	0.03
I4WG83	0.26	-
I4WG92	0.13	0.03
I4WGK8	0.05	0.45
I4WGK9	1.02	0.17
**I4WH42**	-	0.16
I4WH59	0.08	0.03
**I4WHT8**	0.06	0.16
I4WJA7	-	0.06
I4WJS3	0.28	0.36
I4WJU4	0.15	0.10
**I4WM37**	0.10	-
**I4WMC4**	-	0.14
I4WMK6	0.50	0.67
I4WN45	0.15	-
**I4WN49**	0.21	-
**I4WNI6**	-	0.14
**I4WNX0**	-	0.01
I4WPE8	0.15	0.14
**I4WPL8**	0.21	-
I4WPM4	0.25	-
I4WQL9	0.08	0.13
**I4WQS0**	0.08	-
I4WRB7	0.59	0.35

I4WRC0	0.38	0.26
**I4WRF9**	0.64	0.14
I4WRK1	-	0.06
I4WRN7	-	0.02
**I4WRW1**	-	0.04
I4WRX1	0.69	1.28
I4WSB0	0.14	0.08
I4WSB4	-	0.11
I4WSM6	0.01	0.01
I4WSW9	0.07	-
I4WU94	0.19	-
**I4WUA7**	0.28	-
I4WUC4	0.44	0.16
I4WUE0	0.28	-
I4WYZ8	0.39	-

[a] Data are the mean of two replicates.

[b] Proteins in bold characters have a predicted intracellular localization.

[c] Protein not detected or protein identification not considered valid.

Table S3 Proteins produced by *Myxococcus xanthus* DK 1622 during growth in minimal medium supplemented with suberin at day 5 and day 25

Uniprot accession number	Putative function	Abundance (NSpC[a])	
		5 days	25 days
**Amino acid metabolism**			
Q1D8A6	penicillin acylase	0.18	0.11
Q1D5W7	peptidase	0.27	0.03
**Q1D4C2**[b]	Glu/Leu/Phe/Val dehydrogenase	0.36	0.04
Q1D7P1	peptidase	0.24	-[c]
**PNP**	Glu/Leu/Phe/Val dehydrogenase	0.28	-
Q1D9F6	peptidase	0.30	-
Q1DBU7	peptidase	0.29	0.14
**Q1D215**	aminotransferase	0.22	0.22
**Q1DAS8**	prolyl endopeptidase	0.22	0.04
**Q1DAR8**	Xaa-Pro aminopeptidase	0.19	0.04
**Q1CXD4**	peptidase	0.18	0.23
**Q1DAQ9**	Cys/Met metabolism PLP-dependent enzyme	0.19	0.11
Q1DBU8	peptidase	0.22	0.08
**Q1D7X2**	glycine dehydrogenase	0.16	0.05
**Q1CY03**	peptidase	0.15	-
Q!CZK0	pptidase	0.13	-
Q1DD17	peptidase	0.27	0.20
**Q1DC00**	cysteine synthase	0.29	0.39
**Q1CZV8**	4-hydroxy-tetrahydrodipicolinate synthase	0.21	-
**Q1D6Z0**	aspartate aminotransferase	0.12	-
Q1D7L0	peptidase	0.15	-
**Q1D4A7**	urocanate hydratase	0.24	-
**Q1D827**	branched-chain amino acid aminotransferase	0.13	-
**Q1CWK6**	peptidase	0.26	-
Q1CZV3	peptidase	0.19	0.07
**GLYA**	serine hydroxymethyltransferase	0.18	-
**Q1D3P1**	2-oxoisovalerate dehydrogenase	0.19	0.24
Q1D9S1	aminopeptidase	0.10	0.10
Q1DER8	serine protease	0.16	0.05
**METK**	S-adenosylmethionine synthase	0.11	-
Q1D5T1	peptidase	0.11	-
**Q1DEW1**	peptidase	0.18	-
**Q1D3P0**	2-oxoisovalerate dehydrogenase	0.10	-
**HUTH**	histidine ammonia-lyase	0.08	-
Q1CWF2	peptidase	0.09	-
Q1D6F2	peptidyl-dipeptidase	0.14	-
**Q1DDV0**	glutamine synthetase	0.10	0.07
**Q1DAV7**	peptidase	0.13	0.02
**Q1DFN7**	peptidase	0.15	0.21

**SAHH**	adenosylhomocysteinase	0.12	-
Q1DDP8	peptidase.	0.11	-
**Q1CZZ5**	1-pyrroline-5-carboxylate dehydrogenase	0.12	-
**Q1D2E3**	peptidase	0.13	-
**Q1D4V2**	alanine dehydrogenase	0.11	-
**Q1D2K4**	aminopeptidase	0.07	-
Q1CZD2	zinc metalloprotease	0.10	-
**Q1CYE6**	arginine N-succinyltransferase	0.05	-
**CARB**	carbamoyl-phosphate synthase	0.09	-
**HSLU**	ATP-dependent protease	0.10	0.17
**Q1CYQ0**	fumarylacetoacetate hydrolase	0.13	0.13
Q1DBS1	chymotrypsin	0.10	-
F8CLH1	aspartate-semialdehyde dehydrogenase	0.03	-
Q1D9N1	peptidase	0.05	-
Q1DEY9	dipeptidyl peptidase	0.05	-
Q1DAK3	peptidase	0.08	-
**ASTB**	N-succinylarginine dihydrolase	0.06	-
Q1DA13	peptidase	0.06	-
**Q1DAQ3**	cystathionine beta-synthase	0.06	-
**Q1D625**	peptidase	0.05	-
Q1D624	peptidase	0.05	-
Q1DG40	peptidase	0.06	-
**Q1D459**	L-erytro-3,5-diaminohexanoate dehydrogenase	0.02	-
Q1DD72	peptidase	0.02	-
Q1D4R7	peptidase	0.07	-
Q1D6K7	chorismate synthase	0.15	-
Q1CY62	protease	0.04	-
**Q1D3A8**	L-lysine 2,3-aminomutase	0.04	-
Q1CW90	metallopeptidase	0.04	-
Q1CY13	peptidase	0.03	0.09
Q1CYP7	serine protease	0.02	-
Q1DCI3	gamma-glutamyltranspeptidase	0.05	-
**Q1DBP3**	deoxyhypusine synthase family protein	0.03	-
Q1DDS8	protease	0.03	-
Q1D013	glutamate dehydrogenase	0.03	-
A0A0H4W WS1	sarcosine oxidase	0.02	-
Q1DA16	peptidyl-dipeptidase	0.03	-
**Q1D3D3**	dihydrodipicolinate reductase	0.05	-
**DAPB**	4-hydroxy-tetrahydrodipicolinate reductase	0.04	-
**Q1DDD9**	Glu/Leu/Phe/Val dehydrogenase	0.02	-
**Q1D1D4**	peptidase	0.03	0.10
**Q1DCD4**	L-allo-threonine aldolase	0.02	-
**HGD**	homogentisate 1,2-dioxygenase	-	0.13
**Q1D188**	peptidase	-	0.04
Q1D6G9	peptidase	-	0.09

Q1D7W5	aspartate-semialdehyde dehydrogenase	-	0.03
**Q1CYR4**	serine protease	-	0.06
**Q1DFN6**	aminotransferase	-	0.03
Q1D8P6	peptidase	-	0.03
Q1DE55	peptidase	-	0.02
Q1CZR8	peptidase	-	0.04
Q1D8L5	protease	-	0.07
**PAND**	aspartate 1-decarboxylase	-	0.08
**SERC**	phosphoserine aminotransferase	-	0.11
Q1DEW6	peptidase		
**Carbohydrate metabolism**			
**Q1DCV2**	phosphoenolpyruvate carboxykinase	0.22	-
**Q1D5Z8**	fructose-bisphosphate aldolase	0.34	0.06
**Q1D8J1**	glyceraldehyde-3-phosphate dehydrogenase	0.28	0.43
**Q1CVV7**	transketolase	0.13	-
ENO	enolase	0.23	0.05
**TAL**	transaldolase	0.29	-
**Q1DDQ9**	glucose-6-phosphate 1-dehydrogenase	0.17	-
**TPIS**	triosephosphate isomerase	0.26	0.09
Q1CY18	gluconolaconase	0.12	0.11
**Q1D1J6**	GDP-mannose 4,6-dehydratase	0.11	-
Q1DDR1	6-phosphogluconate dehydrogenase	0.10	-
**G6PI**	glucose-6-phosphate isomerase	0.09	0.16
**Q1D0G8**	phosphoglucomutase/phosphomannomutase	0.04	-
Q1CXL1	glucose/sorbosone dydrogenase	0.08	0.04
**Q1D6M2**	UDP-glucose 4-epimerase	0.07	-
**Q1D8J0**	phosphoglycerate kinase	0.06	-
**Q1D6L5**	pyruvate kinase	0.05	-
**Q1CYU9**	pyruvate kinase	0.03	-
**Q1DCE1**	UTP-glucose-1-phosphate uridylyltransferase	0.05	-
Q1D3S1	chitinase	0.05	-
**Q1DDR0**	6-phosphogluconolactonase	0.04	-
**GLGA**	glycogen synthase	0.02	-
**Cell wall, membrane and envelope biogenesis**			
Q1DDQ0	lipoprotein	0.17	0.17
Q1CVX7	lipoprotein	0.23	0.03
Q1CZE8	outer membrane protein	0.23	0.13
MLPA	lipoprotein MlpA	0.20	0.20
Q1D8U6	lipoprotein	0.15	0.14
Q1D0K1	lipoprotein	0.50	0.28
Q1DD51	lipoprotein	0.10	-
Q1D8L4	lipoprotein	0.26	0.17
Q1CXU8	lipoprotein	0.12	0.08
**Q1CYZ9**	Kelch motif/HYR domain protein	0.09	0.06
Q1CWA7	outer membrane protein	0.26	-
Q1DFL0	cell surface protein	0.23	0.20

Q1CZ46	secretion system protein	0.32	-
**Q1CY27**	cell surface protein	0.08	0.06
Q1CY79	lipoprotein	0.32	-
Q1D364	outer membrane lipoprotein	0.12	0.08
Q1D6V9	lipoprotein	0.18	0.04
Q1CXU6	lipoprotein	0.14	0.10
Q1D9N0	cell surface protein lipoprotein	0.18	0.08
Q1CWM1	lipoprotein	0.18	-
Q1D0H4	lipoprotein	0.17	-
Q1DCM4	lipoprotein	0.11	0.11
Q1DE07	chemotaxis protein MotB	0.22	-
Q1D8J2	N-acetylmuramoyl-L-alanine amidase	0.05	0.02
**Q1CY59**	stomatin-like domain-containing protein	0.14	-
**Q1D5A9**	stomatin-like domain-containing protein	0.16	-
Q1D813	LysM domain protein	0.12	-
Q1D9B4	outer membrane protein	0.13	0.05
**Q1CWS3**	chemotaxis protein MotB	0.16	-
Q1CWB1	lipoprotein	0.03	0.03
Q1D1K7	outer membrane protein	0.15	-
Q1D3C1	outer membrane protein	0.13	0.07
Q1DDE9	lipoprotein	0.12	-
Q1CXN6	lipoprotein	0.18	0.18
Q1D049	Ig-like domain protein	0.02	0.01
**Q1D805**	membrane protein	0.05	-
Q1CVX8	lipoprotein	0.13	-
Q1DEJ6	lipoprotein	0.05	0.02
Q1D227	Tol-pal system protein YbgF	0.11	-
**Q1D729**	L-seryl-tRNA(Sec) selenium transferase	0.07	-
Q1DC33	lipoprotein	0.13	-
Q1D638	lipoprotein	0.11	-
Q1D917	outer membrane protein	0.13	0.11
Q1D3F5	pilus assembly protein	0.14	-
Q1D122	lipoprotein	0.18	0.16
Q1DE38	lipoprotein	0.06	-
Q1D139	outer membrane protein	0.09	0.07
Q1DDT8	outer membrane protein	0.09	0.11
Q1CYN2	lipoprotein	0.13	0.08
Q1DDJ2	type IV pilus assembly protein	0.02	-
Q1DA87	lipoprotein	0.05	-
Q1DCK1	pilus assembly protein	0.02	0.01
Q1D0G9	outer membrane protein	0.06	-
Q1DAK4	OmpA domain protein	0.03	-
**Q306N4**	type IV secretion protein PilO	0.07	-
Q1DFQ7	lipoprotein	0.16	-
Q1D1X6	OmpA domain protein	0.05	-
Q1D218	OmpA family protein	0.12	-

Q1D811	MotA/TolQ/ExbB proton channel protein	0.05	-
**Q1D383**	BamA	0.02	-
**Q1D0S5**	UDP-N-acetylmuramyl-tripeptide synthetase	0.05	-
**Q1DD080**	pilus assembly protein PilM	0.04	-
Q1D427	outermmebrane protein	0.09	-
Q1CYZ8	OmpA domain protein	0.04	-
Q1D314	surface cell protein	0.01	0.02
Q1D991	lipoprotein	0.03	-
**Q1D2C6**	OmpA domain protein	0.03	0.10
Q1D1M7	collagen triple helix repeat protein	0.05	0.09
Q1CWC6	lipoprotein	0.06	0.04
Q1D3M7	membrane protein	0.07	-
Q1D1J2	lipoprotein	0.08	-
Q1D8L3	lipoprotein	0.04	0.04
Q1DAX3	lipoprotein	0.05	-
**Q1DAE0**	lipoprotein	0.01	0.02
Q1D6F4	lipoprotein	0.05	-
**Q1DE67**	lipoprotein	0.02	0.03
Q1D0I5	lipoprotein	0.02	-
Q1D191	cell surface protein	-	0.21
Q1CVK5	Ig domain protein	-	0.04
Q1D8Z4	outer membrane lipoprotein	-	0.06
Q1D0T6	OmpA domain protein	-	0.01
Q1D9T7	lipoprotein	-	0.04
Q1DE25	lipoprotein	-	0.07
Q1D271	Ig domain protein	-	0.02
Q1D7R7	lipoprotein	-	0.01
Q1D646	lipoprotein	-	0.03
Q1D7R8	lipoprotein	-	0.02
**Q1D303**	type IV secretion system protein	-	0.06
Q1CXP7	lipoprotein	-	0.02
**Q1CXB9**	lipoprotein	-	0.07
A0A0H4W XC7	outer membrane protein assembly factor	-	0.02
Q1D9P7	Omp A domain protein	-	0.06
**Energy production and conversion**			
**ATPB**	ATP synthase	0.43	0.11
**Q1D4M9**	dihydrolipoyl dehydrogenase	0.38	0.09
**Q1CZF9**	molybdopterin oxidoreductase	0.20	0.07
**ATPA**	ATP synthase	0.38	0.11
**Q1D6L0**	citrate synthase	0.27	0.14
**Q1D8Y7**	pyruvate dehydrogenase	0.34	0.04
**Q1D8Y8**	pyruvate dehydrogenase	0.26	-
MDH	malate dehydrogenase	0.32	0.09
Q1D6J1	succinate dehydrogenase	0.22	0.04
Q1DCK3	aconitate hydratase	0.14	0.02

**Q1CYR1**	isocitrate dehydrogenase	0.31	0.08
**Q1CZK3**	2-oxoglutarate dehydrogenase	0.10	0.01
**Q846U1**	adventurous gliding motility protein	0.21	-
SUCC	succinyl-CoA ligase	0.29	-
**Q1D8Y6**	pyruvate dehydrogenase	0.16	-
**Q1D4N1**	alpha keto acid dehydrogenase	0.26	-
**ATPG**	ATP synthase	0.26	-
Q1D1C4	cytochrome c	0.12	0.07
**Q1CZK2**	2-oxoglutarate dehydrogenase	0.15	-
**Q1CZX3**	pyruvate dehydrogenase	0.14	-
Q1D4Q0	CyaE	0.12	-
Q1CZF6	cytochrome c family protein	0.20	-
**Q1D6I8**	succinyl-CoA ligase	0.18	-
Q1D6J0	succinate dehydrogenase	0.16	-
**ATPD**	ATP synthase	0.25	-
**NUOD**	NADH-quinone oxidoreductase	0.10	-
**Q1D2C8**	aldehyde dehydrogenase	0.06	-
Q1D856	NADH-quinone oxidoreductase	0.16	-
**NUOI**	NADH-quinone oxidoreductase	0.12	-
**Q1D8S8**	NADH dehydrogenase I	0.06	-
**Q1CWX3**	aldehyde dehydrogenase	0.04	-
**Q1DA35**	2-nitropropane dioxygenase	0.07	0.12
**NUOB**	NADH-quinone oxidoreductase	0.05	-
Q1CZG0	cytochrome c	0.12	-
**Q1DDC7**	NADH dehydrogenase	0.14	-
Q1D5X3	PQQ enzyme repeat domain protein	0.07	-
Q1CW00	short chain dehydrogenase	0.05	-
**Q1DDC8**	NADH dehydrogenase	0.05	-
**ATPF**	ATP synthase	0.12	-
**Q1DDE1**	succinate dehydrogenase	0.06	-
**Q1CZF2**	cytochrome c	0.03	-
**Q1D6Y7**	oxidoreductase	-	0.04
**Lipid metabolism and ketogenesis**			
Q1CWS1	lipase	0.31	0.21
**Q1D4F3**	patatin-like phospholipase	0.35	0.13
Q1CYB4	acetyl co-enzyme A carboxylase carboxyltransferase	0.37	0.16
**Q1D030**	acetyl-coenzyme A synthetase	0.19	0.06
**Q1D5V0**	acetyl-CoA acetyltransferase	0.33	0.10
**Q1D9B8**	long-chain-fatty-acid--CoA ligase	0.20	-
Q1D233	3-hydroxyacyl-CoA dehydrogenase	0.17	-
**Q1D5Y2**	carboxyl transferase domain protein	0.19	-
Q1D009	YceI-like family protein	0.27	0.14
**Q1D3D6**	acyl-CoA dehydrogenase	0.13	0.12
**BKT**	beta-ketothiolase	0.17	-
**Q1D5U4**	acyl-CoA dehydrogenase	0.10	-
**Q1D984**	acetyl-coenzyme A synthetase	0.06	-

**Q1DDA0**	propionyl-CoA carboxylase	0.07	0.02
**Q1DFT0**	3-oxoacyl-[acyl-carrier protein] reductase	0.21	-
**Q1D0B9**	acetyl-CoA carboxylase. biotin carboxylase	0.10	-
**Q1D340**	malonyl CoA-acyl carrier protein transacylase	0.11	-
**Q1D5U1**	3-hydroxyacyl-CoA dehydrogenase	0.18	-
**Q1D5U2**	enoyl CoA dehydratase	0.13	-
**Q1D4E4**	acyl-CoA dehydrogenase	0.07	-
**Q1D0T9**	acetyl CoA carboxylase	0.03	-
**Q1D555**	acetyl-coenzyme A carboxylase carboxyl transferase	0.06	-
**Q1D234**	acetyl-CoA acetyltransferase	0.05	-
Q1CZK4	enoyl-[acyl-carrier-protein]	0.07	-
**Q1DDA2**	propionyl-CoA carboxylase	0.04	-
Q1D343	3-oxoacyl-[acyl-carrier-protein] synthase	0.07	-
**Q1D964**	acyl-CoA hydrolase	0.08	-
Q1CYM5	medium-chain fatty acid-CoA ligase	0.03	-
**A0A0H4W WQ8**	acyl-CoA dehydrogenase	0.02	-
**Q1D5Y4**	3-hydroxybutyryl-CoA dehydratase	0.05	-
**Q1D8V2**	acetyl-coenzyme A carboxylase carboxyl transferase	0.05	-
**Q1D5Y1**	acyl-CoA dehydrogenase	0.04	-
**Q1D5W1**	patatin-like phospholipase family protein	0.01	-
**Q1D5V2**	3-oxoacid CoA-transferase family	0.07	-
**Q1D855**	long-chain-fatty-acid--CoA ligase	0.02	-
**Q1D566**	acyltransferase	0.02	-
**Q1CZW5**	acyl-CoA dehydrogenase	0.02	0.02
**Q1D003**	beta-ketothiolase	-	0.13
**Replication, transcription, translation and DNA repair**			
**EFTU1**	elongation factor Tu	0.62	-
**RPOB**	DNA-directed RNA polymerase	0.14	-
Q1CYZ6	cyclic nucleotide-binding domain protein	0.26	-
**Q1DAM8**	transcription termination/antitermination protein NusA	0.27	-
**RPOC**	DNA-directed RNA polymerase	0.10	-
**RPOA**	DNA-directed RNA polymerase	0.22	-
Q1DC39	DNA topoisomerase	0.36	-
Q1CYZ5	cyclic nucleotide-binding domain protein	0.15	-
**Q1D015**	ATP-dependent helicase	0.10	-
**AGLZ**	adventurous-gliding motility protein	0.06	-
**TPS**	development-specific protein	0.21	-
**EFTS**	elongation factor	0.25	-
**Q1D9U0**	glutamine--tRNA ligase	0.12	-
**Q1D8J8**	OB-fold nucleic acid binding domain protein	0.11	-
**Q1DDC0**	response regulator receiver domain protein (CheY-like)	0.11	-
**SYP**	proline--tRNA ligase	0.10	-
**L7UF14**	ribonuclease	0.03	-
**SYM**	methionine--tRNA ligase	0.06	-
SYT	threonine--tRNA ligase	0.07	-

**Q1CWL9**	5'-nucleotidase	0.06	-
**Q1D5U8**	SSU ribosomal protein S1p	0.07	-
**IF2**	translation initiation factor IF-2	0.04	-
**Q1D1Z2**	exodeoxyribonuclease III	0.10	-
**RS2**	30S ribosomal protein	0.12	-
Q1D0I2	PspA/IM30 family protein	0.14	-
**EFG1**	elongation factor G 1	0.04	-
**Q1DCG4**	transcriptional regulator	0.08	-
**Q1DEC3**	signal transduction protein	0.04	-
Q1DFW6	methyl-accepting chemotaxis protein	0.06	-
**SYS**	serine--tRNA ligase	0.02	-
**Q1D7Q3**	DNA-binding protein FruA	0.12	-
**Q1CZ22**	isoleucine--tRNA ligase	0.03	-
**SYA**	alanine--tRNA ligase	0.04	-
**RL9**	50S ribosomal protein L9	0.13	-
**SYN**	asparagine--tRNA ligase	0.05	-
**TIG**	trigger factor	0.05	-
Q1D293	50S ribosomal protein L25	0.13	-
**Q1D5D5**	tRNA pseudouridine synthase	0.03	-
**SYGB**	glycine--tRNA ligase	0.03	-
IF3	translation initiation factor IF-3	0.07	-
**Q1D1I5**	phosphohistidine phosphatase SixA	0.14	-
**Q1D7R1**	DNA polymerase I	0.02	-
**RL10**	50S ribosomal protein L10	0.08	-
**Q1DGB6**	exoribonuclease	0.04	-
**RRF**	ribosome-recycling factor	0.10	-
**Q1D4V8**	gliding motility regulatory protein	0.02	-
**Q1DFR3**	transcriptional regulator	0.19	-
**Q1D3Z2**	valine--tRNA ligase	0.01	-
**Q1D5P9**	tryptophanyl-tRNA synthetase	0.05	-
**Q1D3Z1**	response regulator	0.04	-
**Q1D3C3**	aspartyl-tRNA synthetase	0.01	-
**RL2**	50S ribosomal protein L2	0.05	-
**Q1DEC4**	histidine kinase/response regulator	0.01	-
Q1DAU4	30S ribosomal protein S9	0.10	-
**Q1D3G7**	histidine kinase/response regulator	0.02	-
Q1D8Z3	5'-nucleotidase family protein	0.02	0.02
Q1D668	methyl accepting chemotaxis protein	0.03	-
Q1D087	translation elongation factor	0.05	-
**Q1D0P8**	transcription termination factor Rho	0.02	-
**EFTU2**	elongation factor Tu 2	-	0.23
**Q1DDE2**	single-stranded DNA-binding protein	-	0.11
**Stress and defense mechanisms**			
**CH602**	chaperonin	0.49	0.11
**Q1D7G6**	DSBA-like thioredoxin domain protein	0.47	0.16
**CH601**	chaperonin	0.47	0.19

**Q1DC07**	alkyl hydroperoxide reductase	0.62	0.14
Q1D2K1	thioredoxin domain-containing protein	0.41	0.08
**Q1D7I0**	chaperone protein DnaK	0.21	0.03
**Q1D6H4**	encapsulating protein for peroxidase	0.23	0.20
Q1D2Y6	superoxide dismutase	0.23	0.16
Q1D024	superoxide dismutase	0.20	-
**Q1D5A0**	Lon protease	0.05	-
**B8QZT3**	chaperone HtpG	0.03	-
F8CP79	DSBA-like thioredoxin	0.03	-
**A0A0F7E2A3**	chaperonin	0.18	-
**Q1D2Z9**	chaperone ClpB	0.02	-
**Q1D902**	thioredoxin	0.25	0.13
**Q1D0R6**	chaperone	0.09	-
**Q1DC09**	Dps family protein	0.11	-
**Q1CWM0**	glutathione peroxidase	0.10	-
**Q1D1X3**	thioredoxin domain-containing protein	0.04	-
**Q1CWQ0**	peptide methionine sulfoxide reductase	0.06	-
**Q1D8R6**	OsmC/Ohr family protein	0.07	-
Q1D819	HtrA protease/chaperone protein	0.02	-
**Q1CWL7**	S-formylglutathione hydrolase	-	0.10
**Transport, secretion and efflux**			
Q1DCB6	TonB-dependent receptor	0.40	0.27
**Q1CX48**	TonB-dependent receptor	0.45	-
Q1CWS0	long fatty acid chain transporter	0.35	0.20
Q1D031	porin	0.24	0.14
**Q1D365**	TonB-dependent receptor	0.22	0.18
Q1DBN3	TonB family protein	0.18	-
Q1D2E5	TonB dependent receptor	0.23	0.05
Q1D9E0	secretion pathway protein	0.18	0.04
Q1D2D8	efflux transporter	0.30	0.27
**Q1D304**	type V1 secretion protein	0.22	0.08
Q1DCQ0	TonB dependent receptor	0.16	-
**Q1CYB8**	MotA/TolQ/ExbB proton channel family protein	0.22	0.16
Q1DCG9	PBS lyase HEAT-like repeat protein	0.13	-
Q1D4S2	outer membrane efflux protein	0.17	0.13
**Q1CX94**	MotA/TolQ/ExbB proton channel protein	0.23	0.12
Q1DE39	TonB family protein	0.10	0.03
Q1CVN2	efflux protein	0.18	0.05
**Q1DCB8**	MotA/TolQ/ExbB proton channel protein	0.21	-
**A176_005531**	Oar protein	0.01	-
Q1CXU1	branched chain amino acid ABC transporter	0.19	0.21
Q1DDZ2	apolipoprotein-containing domain	0.10	-
Q1D4F4	arsenical pump-driving ATPase	0.13	-
**Q1D697**	iron-chelator utilization protein	0.17	0.13

	Q1DB13	porin	0.11	0.08
	Q1D3P6	TonB dependent receptor	0.05	-
	Q1CVZ3	porin	0.12	0.10
	Q1DEY6	TonB-dependent receptor	0.05	0.02
	**SECA**	translocase SecA	0.04	-
	Q1D9H3	signal peptide peptidase SppA	0.06	-
	**Q1DFV8**	efflux pump	0.05	-
	Q1DEU2	phosphate-selective porin	0.08	-
	Q1DCV4	ABC transporter	0.08	0.08
	Q1DE87	iron ABC transporter	0.09	-
	**Q1DEU5**	ABC transporter	0.04	-
	Q1D5I8	efflux transporter	0.08	0.02
	**Q1D099**	twitching motility protein	0.09	-
	**Q1D2U8**	TonB domain protein	0.05	-
	**Q1DD62**	amino acid transporter	0.07	0.04
	Q1DFZ8	WD domain G-beta repeat/PBS lyase HEAT-like repeat protein	0.01	-
	**Q1DEV0**	ABC transporter	0.03	-
	Q1CWK2	cysteine-rich secretory protein	0.14	-
	TOLB	TolB	0.07	-
	Q1DEG8	ferric siderophore ABC transporter	0.06	-
	**Q1D696**	siderophore biosynthesis aminotransferase	0.05	0.03
	Q1DCP7	hemin ABC transporter	0.05	-
	**Q1DCC0**	ExbD/TolR transport protein	0.11	-
	Q1D7R4	protein transporter	0.04	-
	Q1CZ64	efflux transporter	0.05	-
	**Q1CYB7**	transport energizing protein	0.17	-
	**Q1D3B5**	preprotein translocase YajC	0.17	-
	Q1D521	ABC transporter	0.05	-
	Q1DCB7	TonB	0.08	-
	Q1D8I1	Nit/TauT transport system	0.05	-
	Q1CVG1	insertase YidC	0.02	-
	**Q1D3Z8**	polyketide cyclase/dehydrase and lipid transport	0.09	-
	Q1CY23	TonB-dependent receptor	0.02	-
	Q1D5Z6	phospholipid transport system	0.07	-
	Q1D1X8	PBS lyase HEAT repeat-like domain protein	0.03	-
	A0A0H4WT21	arsenical pump-driving ATPase	0.01	-
	**Q1D9M3**	ABC transporter	0.04	-
	Q1CVX9	TonB-dependent receptor	0.01	-
	**Q1D0D3**	MotA/TolQ/ExbB proton channel protein	0.04	-
**Other functions**				
	Q1CWK0	peptidyl-prolyl cis-trans isomerase	0.52	0.31
	Q1D9I3	5'-nucleotidase family protein	0.31	0.12
	Q1D3W0	phage tail sheath protein	0.20	0.30
	Q1DF51	myo-inositol-1-phosphate synthase	0.28	-

Q1DCH7	alkaline phosphatase	0.17	0.14
Q1D0E3	aspartyl beta-hydroxylase	0.22	0.08
Q1DEX1	NAD dependent epimerase/dehydratase	0.18	-
**Q1CVJ5**	bactofilin	0.63	-
**NDK**	nucleoside diphosphate kinase	0.66	-
Q1D4V7	frizzy aggregation protein	0.24	0.07
Q1DD37	peptidyl-prolyl cis-trans isomerase	0.23	-
**Q1D4E5**	phosphodiesterase/nucleotide pyrophosphatase family protein	0.19	-
**Q1DCD9**	glutathione S-transferase	0.28	0.09
**Q1DC52**	adenosine deaminase	0.19	-
**GLPK**	glycerol kinase	0.17	-
**Q1D458**	feruloyl esterase	0.15	0.19
**Q1D5W4**	iInosine-5'-monophosphate dehydrogenase	0.11	0.05
CGLB	CglB	0.11	0.05
**PURA**	adenylosuccinate synthetase	0.12	-
**Q1DA41**	B12 binding domain/ArgK/methylmalonyl-CoA mutase family protein	0.05	-
**Q1D440**	methyl transferase	0.09	-
**Q1CVR5**	vegetative protein	0.21	-
Q1D1J7	phytase	0.11	-
Y1394	beta lactamase	0.14	-
**Q1CYB9**	oxidoreductase	0.14	-
Q1D6F6	metallophosphoesterase	0.12	0.03
**Q1DDZ8**	glutathione S-transferase	0.22	-
**Q1D5X7**	peptidyl-prolyl cis-trans isomerase	0.19	-
Q1DFJ0	oxidoreductase	0.14	-
**Q1D7G7**	dihydrofolate reductase	0.21	0.29
**Q1D3H2**	bactofilin	0.16	0.06
**Q1DG65**	amidohydrolase	0.01	-
**Q1D4E8**	polyketide synthase	0.02	-
**L7U7N0**	serine-threonine protein kinase	0.05	-
**PYRF**	orotidine 5'-phosphate decarboxylase	0.10	-
Q1CWR7	oxidoreductase	0.07	-
**Q1DCF0**	NAD-dependent epimerase/dehydratase	0.13	-
PYRG	CTP synthase	0.05	-
**Q1D418**	serine/threonine protein kinase	0.03	-
**PYRB**	aspartate carbamoyltransferase	0.09	-
Q1D2U6	adventurous gliding motility protein AglT	0.06	-
**Q1DG46**	amidohydrolase	0.04	-
**DEOD**	purine nucleoside phosphorylase	0.13	0.13
**PUR4**	phosphoribosylformylglycinamidine synthase	0.03	-
**Q1CYZ7**	terpene synthase	0.04	-
**Q1CVY7**	oxidoreductase	0.07	-
**Q1D4M7**	DofB	0.15	-
**Q1CXA7**	peptidyl-prolyl cis-trans isomerase	0.23	-

**Q1D3V9**	phage tail protein	0.16	0.38
**Q1D025**	methyl transferase	0.03	-
**Q1CVM7**	glycosyl transferase	0.04	-
Q1DA70	pirin family protein	0.05	-
Q1D2V2	adventurous gliding motility protein AgmX	0.04	-
**Q1D0H5**	pyrimidine-nucleoside phosphorylase	0.07	-
Q1D669	biotin synthase	0.10	-
**Q1D7X4**	aminomethyltransferase	0.06	-
Q1D3H8	L-aspartate oxidase	0.04	-
**Q1CXJ4**	coproporphyrinogen oxidase	0.06	-
**Q1DDY8**	glutathione S transferase	0.06	-
Q1D806	adventurous gliding motility protein AglU	0.04	-
Q1D689	2,3-dihydro-2,3-dihydroxybenzoate dehydrogenase	0.06	-
**Q1CZT4**	acetyltransferase	0.15	-
Q1D4E6	FAD-binding domain protein	0.03	-
**A0A0H4X GT4**	quorum sensing signal AsgA	0.05	-
**Q1D4A1**	bifunctional NAD(P)H-hydrate repair enzyme	0.03	-
Q1D9Y5	glutathione-disulfide reductase	0.03	0.05
A0A0H4W UN0	peptidyl-prolyl cis-trans isomerase PpiA	0.02	-
**Q1D6L8**	dihydroorotase	0.03	-
**Q1D2F6**	decarboxylase family protein	0.03	0.08
PSD	phosphatidylserine decarboxylase	0.06	-
Q1CWP9	myxobacterial hemagglutinin	0.04	0.05
**Q1D1B9**	choloylglycine hydrolase family protein	0.05	-
**Q1D965**	HesB/YadR/YfhF family protein	0.18	-
**Q1CYQ7**	glycine cleavage system T protein	0.04	-
**Q1DEX3**	aminotransferase	0.03	-
**Q1D0V7**	Inorganic pyrophosphatase	0.06	-
**Q1D4F6**	polyketide synthase	0.01	-
**Q1CXC7**	late embryogenesis abundant-like protein	0.07	-
**Q1CVW0**	vegetative protein	0.05	-
**Q1D5D1**	pyridine nucleotide-disulphide oxidoreductase	0.03	-
**Q1D7W4**	ferrodoxin	0.17	-
**F8CEV8**	pseudouridine synthase	0.02	-
Q1D910	peptidylprolyl isomerase	0.03	-
**MGLA**	gliding motility protein MglA	0.07	-
**Q1DDR3**	AMP-binding protein	0.02	-
Q1D7M7	xanthine dehydrogenase	0.01	0.03
**Q1D559**	glutathione S-transferase	0.04	-
Q1D161	male sterility protein-like protein	0.03	-
**Q1D7K0**	oxidoreductase	0.03	-
Q1DFD3	endonuclease/exonuclease/phosphatase family protein	-	0.12
**DESS**	development-specific protein S	-	0.16
**Q1DDX6**	amidohydrolase	-	0.01

**Q1CWL6**	S-(hydroxymethyl)glutathione dehydrogenase	-	0.04
**Q1D3U9**	phage tail protein	-	0.02
**Q1D6W6**	3,4-dihydroxy-2-butanone 4-phosphate synthase/GTP cyclohydrolase II	-	0.02
**PNP**	polyribonucleotide nucleotidyltransferase	-	0.03
**Function unknown**			
Q1DBN2		0.28	-
Q1D2V4		0.18	0.21
Q1D5E0		0.23	-
Q1D8Z5		0.27	0.28
Q1D2R6		0.33	0.12
Q1D2U5		0.17	-
Q1CVR0		0.18	0.14
**Q1D1M5**		0.22	0.19
**Q1CZV5**		0.13	0.03
Q1D2E4		0.23	0.16
Q1D9N6		0.09	0.06
Q1CXK5		0.29	0.13
**Q1CZI8**		0.22	-
Q1CXW5		0.14	-
Q1D077		0.33	0.27
Q1D9B3		0.13	-
Q1D840		0.05	-
Q1D6H7		0.19	0.21
Q1D5W5		0.35	-
Q1D3G6		0.15	0.04
Q1D2V0		0.14	0.05
Q1D7L1		0.06	-
Q1D2U9		0.18	0.09
Q1D4F5		0.38	0.19
Q1D959		0.13	0.08
Q1DEA6		0.10	-
Q1D0J9		0.29	0.21
Q1D5R1		0.13	0.10
**Q1D9C8**		0.23	-
Q1CX67		0.10	-
Q1CW98		0.23	0.13
Q1D0K0		0.16	0.18
**Q1D4U1**		0.15	-
Q1DBT9		0.07	0.02
Q1DDR7		0.04	0.03
Q1DEJ3		0.29	0.29
**Q1D4F9**		0.05	-
**Q1V0D2**		0.13	-
Q1CYL8		0.08	-
Q1CX74		0.24	-

**Q1D3V0**	0.07	0.08
**Q1DFZ6**	0.05	-
**Q1DC42**	0.07	-
**Q1D6G2**	0.08	0.01
**Q1D9T8**	0.08	0.09
**Q1DEJ8**	0.08	0.03
Q1D9B5	0.07	0.07
Q1CWJ8	0.05	-
Q1D4R8	0.06	-
Q1DA21	0.24	-
**Q1DFS2**	0.08	-
**Q1D9J1**	0.10	-
**Q1D1K3**	0.05	-
Q1D904	0.25	-
Q1D0U7	0.08	-
**Q1D3U7**	0.06	0.11
Q1D8D6	0.02	0.04
Q1DET2	0.09	-
Q1DCJ4	0.13	0.11
**Q1DBX9**	0.07	-
Q1DDY7	0.12	-
**Q1DEJ9**	0.04	-
Q1D9M7	0.05	0.08
Q1D5D6	0.06	-
**Q1D3A5**	0.09	-
**Q1DF90**	0.13	-
Q1D0E8	0.08	-
Q1D450	0.14	0.09
**Q1D2W3**	0.05	0.08
**Q1D9I8**	0.06	-
**Q1CVW3**	0.12	-
Q1CYX4	0.02	0.05
Q1CZ57	0.05	-
Q1CWY2	0.07	-
Q1DCE2	0.08	-
Q1CXT3	0.07	-
Q1D1Q5	0.06	-
**Q1D3U8**	0.06	0.06
**Q1DDX9**	0.11	-
Q1CZ58	0.04	-
**Q1CZF4**	0.12	-
Q1D615	0.08	-
Q1DEF2	0.11	-
Q1D2J1	0.06	-
Q1D100	0.06	-
**Q1DCA5**	0.12	-

**Q1D417**	0.03	-
**Q1DBU4**	0.08	-
Q1CZ63	0.02	-
**Q1D4R1**	0.02	-
**Q1DFH8**	0.06	-
Q1D4H9	0.08	-
Q1DD93	0.02	-
Q1CYW3	0.03	-
**Q1D1C2**	0.03	-
Q1DB38	0.08	-
**Q1D4U3**	0.02	-
Q1CVK8	0.14	0.23
Q1D2D5	0.09	-
**Q1D6J5**	0.05	-
Q1D992	0.05	-
**Q1DAD6**	0.05	-
**Q1CZF7**	0.05	-
**Q1D8H6**	0.08	-
**Q1D8C0**	0.02	-
Q1CZ50	0.01	-
Q1D1D7	0.02	-
Q1D680	-	0.12
**Q1D3V8**	-	0.06
Q1CXB7	-	0.11
**Q1DA09**	-	0.07
Q1DDQ6	-	0.03
Q1CX13	-	0.06
Q1CVF7	-	0.01

[a] Data are the mean of two replicates.

[b] Proteins in bold characters have a predicted intracellular localization.

[c] Protein not detected or protein identification not considered valid.
